# Glutamine Supplementation in Sick Children: Is It Beneficial?

**DOI:** 10.1155/2011/617597

**Published:** 2011-11-14

**Authors:** Elise Mok, Régis Hankard

**Affiliations:** ^1^INSERM Centre D'Investigation Clinique 802, Centre Hospitalier Universitaire de Poitiers, 86021 Poitiers Cedex, France; ^2^Pédiatrie Multidisciplinaire-Nutrition de l'Enfant, Centre Hospitalier Universitaire de Poitiers, 86021 Poitiers Cedex, France; ^3^Child Health Clinical Research Centre, The Montréal Children's Hospital, McGill University Health Centre, Montréal, QC, Canada H3H 1P3

## Abstract

The purpose of this review is to provide a critical appraisal of the literature on Glutamine (Gln) supplementation in various conditions or illnesses that affect children, from neonates to adolescents. First, a general overview of the proposed mechanisms for the beneficial effects of Gln is provided, and subsequently clinical studies are discussed. Despite safety, studies are conflicting, partly due to different effects of enteral and parenteral Gln supplementation. Further insufficient evidence is available on the benefits of Gln supplementation in pediatric patients. This includes premature infants, infants with gastrointestinal disease, children with Crohn's disease, short bowel syndrome, malnutrition/diarrhea, cancer, severe burns/trauma, Duchenne muscular dystrophy, sickle cell anemia, cystic fibrosis, and type 1 diabetes. Moreover, methodological issues have been noted in some studies. Further mechanistic data is needed along with large randomized controlled trials in select populations of sick children, who may eventually benefit from supplemental Gln.

## 1. Introduction

Glutamine (Gln) is the most abundant amino acid in the muscle and plasma of humans [1]. Although Gln is a nonessential neutral amino acid, it is necessary for optimal growth of mammalian cells in tissue culture [2] and has important physiological functions. Apart from providing nitrogen for protein synthesis, Gln is a precursor for nucleic acids, nucleotides [3], hexosamines [4], the nitric oxide precursor-arginine (Arg) [5], and the major antioxidant-glutathione [4, 6]. Gln is also an important oxidative fuel for rapidly proliferating cells such as those of the gastrointestinal tract [7] and immune system [3], reticulocytes [8], fibroblasts [9], and so on. It plays a central role in nitrogen transport between tissues [10], specifically from muscle to gut, kidney, and liver. In addition to its role as a gluconeogenic substrate in the liver, kidney [11], and intestine [12], Gln is involved in the renal handling of ammonia, serving as a regulator of acid-base homeostasis [13]. Present data also indicate that Gln functions as a signalling molecule [14], particularly under catabolic conditions.

Traditionally Gln is considered a nonessential amino acid, because it is synthesized by most tissue (skeletal muscle being the main producer and storage site) [15]. Gln synthetase catalyzes the terminal step in Gln *de novo* synthesis and is a key enzyme in Gln metabolism [16, 17]. In mammals, Gln synthetase expression is regulated by transcriptional and posttranscriptional mechanisms, that is, increasing Gln synthetase mRNA in response to stress (e.g., glucocorticoids) and regulation of Gln synthetase protein turnover in response to its product (Gln concentrations) [18]. The importance of Gln at the whole body level is highlighted by the report of severe brain malformation resulting in multiorgan failure and neonatal death in 2 unrelated newborns with congenital Gln synthetase deficiency, in whom Gln was largely absent in plasma, urine, and cerebral spinal fluid [19].

Under normal conditions, Gln is released into circulation for consumption by other tissue, whereas during catabolic stress the production of Gln may be insufficient to meet the increased requirements by the gut, immune system/inflammatory cells, liver, and kidneys. Demands are partly met by skeletal muscle proteolysis and release of large amounts of Gln to maintain normal concentrations in the plasma, resulting in depletion of Gln stores. Based on this abundant evidence, Lacey and Wilmore [10] suggested that Gln may become a conditionally essential amino acid for the critically ill.

In paediatrics, several researchers have studied the efficacy of supplemental Gln in premature infants of low birth weight (LBW), who are highly stressed and have low energy and protein reserves [20]. Similar to premature neonates, Gln supplementation may also be beneficial for other childhood conditions including gastrointestinal disease, malnutrition, cancer, severe burns/trauma as well as chronic diseases of childhood. However, less data is available on the effects of supplemental Gln in older infants and children with various diseases.

In addition to being sick and highly stressed, children are also in the process of growth and development. Hence, specific research on the role of Gln in pediatric patients is necessary. The main purpose of this manuscript is to provide a critical review of the literature on Gln supplementation in various conditions/illnesses that affect children (from neonates to adolescents). First the proposed mechanisms of Gln action are reviewed in a general context, followed by a detailed description and critique of the clinical research on Gln supplementation in children.

### 1.1. Glutamine Mechanisms of Action

**Figure 1:**
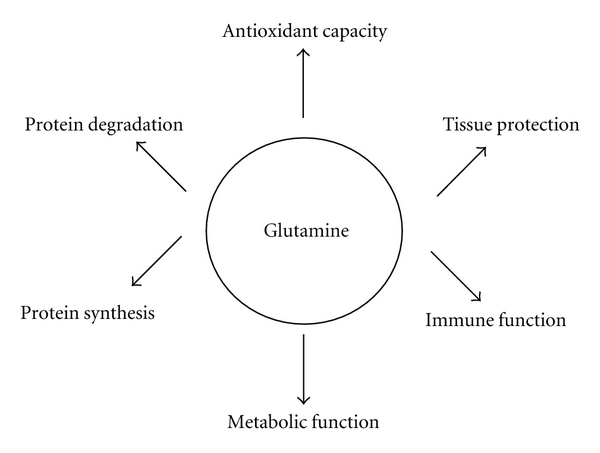
Schematic representation of the mechanism of glutamine action.

While it is well established that Gln is a protein precursor as well as a major fuel and nucleotide substrate for rapidly proliferating cells (e.g., gut and immune system) [3, 7], additional mechanistic data has emerged to explain the apparent benefits of Gln. Gln can regulate the expression of many genes related to metabolism, signal transduction, cell defense, and repair and can activate intracellular signaling pathways [14]. In brief, Gln seems to affect antioxidant capacity, tissue protection, immune, and metabolic function [21] as well as protein synthesis and degradation [14] (Figure 1). The postulated mechanisms remain speculative and are by no means mutually exclusive, since Gln can provoke a number of different effects that interact with one another. 

### 1.2. Antioxidant Capacity

#### 1.2.1. Glutathione

Gln is a precursor of the glutamate (Glu), for glutathione (L-*γ*-glutamyl-L-cysteinyl-glycine) synthesis, an important antioxidant in many cell types [22]. Glutathione is present in the cell in reduced (GSH) and oxidized (GSSG) forms. The ratio of reduced-to-oxidized glutathione is the major regulator of the cellular redox potential that determines the antioxidant capacity of the cell [14, 22, 23]. The effectiveness of glutathione protection in individual tissue depends on the tissue concentration of glutathione as well as the capacity of the tissue to import GSH and to export GSSG [24].

*In vivo* experiments in rats demonstrate that administration of Gln before ischemia/reperfusion injury or surgical manipulation can enhance GSH concentrations and provide protection against oxidative stress in various tissues (e.g., cardiac, intestinal, and lung) [25, 26]. Further, the effectiveness of Gln in preventing liver damage in neonatal sepsis appears to be mediated via glutathione synthesis [27]. In humans, Gln supplementation can attenuate GSH depletion in skeletal muscle following surgical trauma [28].

During critical illness, muscle concentrations of GSH decrease and a change in the redox status occurs, indicative of an elevated GSSG [24]. Moreover, there is a correlation between the concentrations of Gln and GSH [24]. Shifting the GSH/GSSG redox toward oxidizing conditions activates several signaling pathways, such as c-Jun N-terminal kinase (JNK), apoptosis signal-regulated kinase-1 (ASK-1), mitogen-activated protein kinase (MAPK), and the transcription factor nuclear factor-kappaB (NF-*κ*B: a stimulator of the synthesis of proinflammatory cytokines and adhesion molecules) [22, 23, 29]. Evidence also implicates oxidative stress as a potential regulator of NF-*κ*B transactivation by MAPKs (in particular extracellular signal-regulated kinase 1/2 (ERK1/2) and p38) [30] which could lead to increased proteolysis in muscle [31]. Moreover, the cellular redox status seems to be related to the degree of muscle protein degradation [32, 33]. Likewise, there is significant literature on the role of inflammatory cytokines (interleukin-1, -6, tumour necrosis factor-alpha (TNF-*α*)) and muscle wasting [34].

Gln metabolism via entry into the citric acid cycle may allow the activation of malic enzyme which will result in an increase in NADPH [15] and subsequently increase the GSH/GSSG ratio [14]. Administration of Gln leads to an increased ratio of GSH/GSSG and reduces the activity of redox sensitive kinases subsequently preventing NF-*κ*B activation and thus inhibiting the inflammatory response [23].

Experiments from our group in the *mdx* mouse model of muscular dystrophy (a condition associated with severe muscle wasting) showed that *in vivo* Gln administration can reduce GSSG in dystrophic skeletal muscle, hence, increasing the ratio of GSH/GSSG [35]. This was associated with decreased activation of MAPK (ERK1/2) pathway [35]. Similar effects were observed in muscle of control mice; however, the magnitude was less [35]. Thus, in muscle tissue, Gln might affect the cellular redox state involving MAPK pathway.

### 1.3. Tissue Protection

#### 1.3.1. Heat Shock Protein (HSP)

The HSPs serve as molecular chaperones that appear to repair denatured/injured proteins or promote their degradation following irreparable injury. Gln has cell-protective effects, as a potent enhancer of the expression of HSP25, HSP70, HSP72, and heme-oxygenase-1 in cell culture [36], in multiple organs of both stressed and unstressed animal models [37], as well as in humans [38, 39]. However, Gln depletion during the stress response can impair the expression of the major inducible HSP (HSP70), as shown in human lymphocytes [40]. And recent evidence suggests that HSP70 expression is required for Gln's protection against tissue injury and for attenuation of NF-*κ*B activation and proinflammatory cytokine release [41].

In rats, preoperative administration of Gln induces HSP70 expression and attenuates the inflammatory response by regulating nitric oxide synthase (NOS) activity in heart, lung, and liver [42]. Gln is a well-known precursor for Arg [5], which can increase nitric oxide formation as a result of enhanced NOS activity [6]. However, in various models of human intestinal cells, Gln does not further increase nitric oxide production or inducible NOS mRNA following proinflammatory cytokine stimulation [43]. Thus, Gln's effects on NOS activity might be tissue- or condition-specific.

The survival-promoting effects of HSP70 can also be attributed in part to the suppression of apoptosis, since reduced HSP expression in Gln-deprived cells together with their impaired antioxidant capacity may make them more susceptible to apoptosis [29].

#### 1.3.2. Apoptosis

Gln starvation has been shown to induce apoptosis in intestinal epithelial cells [44] and also renders human monocytic cells more susceptible to apoptosis induced by Fas ligand, heat shock, or TNF-*α* stimulation [45]. In HeLa cells, Gln might also suppress ASK-1 and JNK/stress-activated protein kinase (SAPK) activation by Fas ligand [46]. The effect of Gln in delaying spontaneous apoptosis in neutrophils may be mediated by the antioxidant effects of glutathione [47]. Furthermore, Gln may protect activated T cells from apoptosis, partially by upregulating glutathione and Bcl-2 expression and inhibiting Fas [48].

#### 1.3.3. Intestinal Barrier Function

As an important fuel for intestinal tissue and gut-associated lymphoid tissue, Gln may contribute to gut barrier function [49]. Experimental data from neonatal animal models also support the beneficial effects of Gln on gastrointestinal development and function [50–59]. Gln is also involved in the biosynthesis of hexosamines which are important for maintaining gut wall integrity via surface mucin and glycoprotein-forming intracellular tight junctions and thus may protect against bacterial translocation [4, 60].

### 1.4. Immune Function

As a major fuel for immune cells, Gln is known to modulate immune function. More recently, Gln has also been shown to have anti-inflammatory effects, modulating cytokine production, both *in vitro* [36, 61–63] and *in vivo* [41, 51, 64], possibly through decreased NF-*κ*B activation [41, 62], a major transcription factor regulating immune and inflammatory responses. In neonatal mice and rats with experimental NEC, Gln reduces intestinal injury [54, 55], via mechanisms inhibiting inflammatory cytokine release [54].

Gln's anti-inflammatory effects may also be related to enhanced HSP expression [36, 41]. The induction of HSP response can attenuate proinflammatory cytokine release, which in turn depends on the cellular redox potential and consequently is regulated by the intracellular GSH/GSSG ratio [14] (as previously described).

### 1.5. Tissue Metabolic Function

Gln can preserve tissue-metabolic function in stress states. For instance, Gln enhances myocardial tissue-metabolic function after ischemia/reperfusion injury in rats [26]. Gln can also enhance ATP levels in oxidant stressed endothelial cells [65].

#### 1.5.1. Glucose Metabolism

While Gln plays an important role in gluconeogenesis [11, 12, 66], evidence also suggests that Gln can improve insulin sensitivity and glucose disposal in patients suffering from critical illness [21, 67], a condition frequently associated with insulin resistance and subsequent hyperglycemia. Gln also plays a role as a signaling molecule in amino acid- and glucose-stimulated insulin secretion [68]. Interestingly in rats with diet-induced obesity, Gln supplementation induces insulin resistance in adipose tissue and reduces adipose mass, consequently attenuating insulin resistance and activation of JNK and inhibitory kappaB kinase subunit beta in liver and muscle, thus improving insulin signaling [69]. These data suggest that Gln can beneficially influence insulin-dependent glucose metabolism.

### 1.6. Protein Synthesis and Degradation

Gln appears to regulate protein turnover in cultured rat skeletal myotubes, stimulating protein synthesis in stressed myotubes while inhibiting protein degradation in long-lived proteins. This may be related to the increase in HSP70 [70]. There is also abundant literature to suggest that amino acids affect protein turnover via the mammalian target of rapamycin (mTOR) pathway [71, 72].

#### 1.6.1. Protein Synthesis

Amino acids, particularly branched chain amino acids, for example, leucine (Leu) stimulate skeletal muscle protein synthesis via the activation of mTOR which in turn activates p70 ribosomal S6 kinase (p70^S6K^) and dephosphorylates eukaryotic initiation factor 4E-binding protein 1 (4E-BP 1), stimulating translation and protein synthesis [71, 72]. Gln can induce growth and maturation of neonatal rat cardiomyocytes, which is associated with an increase in the mRNAs-encoding contractile proteins and metabolic enzymes via the activation of protein kinase A and mTOR [73]. However, the action of Gln seems to be cell-type specific. For instance, in C2C12 myogenic cells, Gln and Leu have opposite effects on the mTOR pathway [74]. Whereas Leu activates this pathway, Gln inhibits it by decreasing the phosphorylation states of mTOR (on serine (Ser)2448), p70^S6K^, and 4E-BP1, with no effect on protein synthesis [74].

#### 1.6.2. Protein Degradation

The major proteolytic pathways in organs such as the liver, muscle, and intestine include the autophagic/lysosomal (cathepsins), the calcium activated (calpains), and the ATP-ubiquitin-proteosome pathway [14, 34]. The proteosome system (26S) is a highly selective proteolytic pathway [71]. In visceral tissues (e.g., liver), autophagy is, however, the major proteolytic pathway and the only pathway known to be regulated by plasma amino acids (in liver and skeletal muscle) [71, 72]. In autophagic proteolysis, several amino acids have direct regulatory potential, possibly via a plasma membrane amino acid receptor/sensor and subsequent intracellular signaling [71].

Another line of evidence suggests that amino acids activate mTOR pathway which in turn suppresses protein breakdown by the autophagy/lysosomal pathway [71, 72]. Amino acids can also control autophagic lysosomal proteolysis by inhibiting MAPK (ERK1/2) phosphorylation [75]. Gln may cause its antiproteolytic effect through osmotic swelling [76], involving p38 MAPK pathway [77]. An increase in cellular hydration acts as an anabolic signal, whereas cell shrinkage is catabolic, and there is a close relation in the regulation of cell volume, Gln, and protein catabolism [23].

On the other hand in the human gut, enteral Gln may attenuate ubiquitin-dependent proteolysis as demonstrated by decreased ubiquitin mRNA, whereas mRNAs encoding for cathepsins or calpains were not affected [78]. Furthermore, in lung and muscle, Gln can also regulate its own production through a posttranscriptional mechanism in which Gln regulates Gln synthetase protein degradation [16, 17, 79], by facilitating its degradation by the 26S proteosome [18]. Thus, the presence of Gln could have a protein-sparing effect, sparing amino acids for protein accretion [80–82].

### 1.7. Glutamine in the Neonatal Period

Gln is the predominant amino acid supplied to the fetus through the placenta and is specifically suited for its rapid development [83, 84]. While normally present in the enteral diet, Gln has been excluded from parenteral nutrition (PN) because of low solubility and instability in solution. In the first weeks of life, however, premature infants receive most of their nutrients from PN which is Gln-free [85]. The sudden cessation of Gln supply from the mother to premature infants, who are already stressed and undergoing rapid growth, may be detrimental [86]. Whereas plasma Gln concentrations normally increase during the first days of life in newborn infants breastfed *ad libitum* [87], selective amino acid deficiencies have been reported in neonates suffering from acute illness, including reductions in serum Gln and Arg in infants who have necrotizing enterocolitis (NEC) that may predispose them to the illness [88]. It has been suggested that in catabolic conditions premature infants are not able to synthesize sufficient Gln to meet demands, and in these conditions Gln may become a conditionally essential amino acid [10].

### 1.8. Glutamine in Breast Milk

In addition to providing an ideal nutritional composition for the neonate, breast milk contains specific nutrients such as Gln that may influence gastrointestinal development and can modulate immune, metabolic, and inflammatory responses [89]. Deprivation of dietary or endogenously synthesized Gln results in a breakdown in the intestinal epithelium of artificially reared neonatal rats, whereas Gln supplementation may help to maintain intestinal integrity [59].

In extremely low birth weight (ELBW) infants, the beneficial effects of breast milk ingested in the neonatal intensive care unit (NICU) on developmental outcomes at 18 months of age [90] persist at 30 months [91]. While PN is Gln-free, Agostoni et al. [92] reported lower free Gln concentrations in standard infant formulas compared to breast milk collected from 40 healthy lactating mothers after delivery of term infants at age 1 month. And similar to previous reports [93], they observed that glutamic acid and Gln accounted for most of the free amino acids in breast milk [92]. Moreover, heat sterilization of infant formulas can further lower the concentrations of Gln by more than 60% [94]. Thus, suggesting that enrichment of infant formulas with nonprotein nitrogen components (particularly Gln and glutamic acid) could be beneficial. The same group followed 16 healthy exclusively breastfeeding mothers after delivery of term infants and showed that the concentrations of free glutamic acid and Gln increased by 2.5 and 20 fold, respectively, with progressing lactation, representing >50% of total free amino acids by 3 months [95]. To assess the potential influence of gestational age and duration of lactation, Jochum et al. [96] measured the content of free and protein-bound Gln in transitional and mature breast milk of 40 healthy mothers after term and preterm delivery. While the time of delivery had no influence on the free Gln or total Gln concentration, free Gln concentrations increased during maturation of lactogenesis, similar to previous reports [95, 97, 98]. In contrast, total Gln and protein-bound Gln concentrations decreased with the duration of lactation, and this correlated with the decrease in total protein concentration in mature breast milk [96]. These data highlight the need to better define the role of Gln in the neonatal period and the possible benefit of supplemental Gln.

## 2. Materials and Methods

The following sections describe the clinical studies that examined the effects of parenteral and enteral Gln supplementation in premature neonates as well as older infants and children with various diseases. The search methods for identification of studies consisted of searches of PubMed (1966–June 2011). The database was searched using the search term: “glutamine.” The search output was limited with the search filter for ages: all children 0–18 years. There were no language restrictions. References in previous reviews and studies were examined also. The title and abstract of all studies identified by the above search strategy were screened, and the full text for all potentially relevant studies published in English was obtained. The full text of any potentially relevant studies was assessed by the first author. Studies that included only adult participants were excluded. The same author extracted data from the published studies.

## 3. Results and Discussion

### 3.1. Parenteral Glutamine Supplementation in Premature Neonates

#### 3.1.1. Clinical Outcomes

The first evidence to suggest that parenteral Gln appears safe and may be considered a conditionally essential amino acid in premature infants was put forth more than a decade ago by Lacey et al. [99] (Table 1). Although efficacy was not demonstrated in the entire cohort of very low birth weight (VLBW) infants (*N* = 44), subgroup analysis in the infants with birth weight <800 g (*n* = 24) showed that those supplemented with Gln (20% of amino acids) had fewer d on PN (13 versus 21 d, *P* < 0.05), required less time to full feeds (8 versus 14 d, *P* < 0.05), and needed less time on a ventilator (38 versus 47 d, *P* < 0.05) compared to standard isonitrogenous isocaloric PN. The positive results, however, were based on subgroup analyses, followup of recruited infants was incomplete, and intention-to-treat analysis was not performed. Thompson et al. [100] further demonstrated that parenteral Gln may reduce the time to establish full feeds and appears to be well tolerated and safe in a group of 35 ill ELBW neonates randomized to standard PN supplemented with Gln (16% of amino acids) or standard PN containing an isocaloric isonitrogenous amino acid solution. The primary outcome (median d to achieve full enteral nutrition (EN)) was significantly shorter in the Gln group (Gln: 13 d versus control: 21 d, *P* < 0.05), whereas other clinical outcomes (growth, infection, number of episodes of sepsis, NEC, or age at discharge) did not differ. The study, however, did not achieve the calculated sample size of 120 infants. It is also not clear whether groups differed with respect to enteral feeding with either breast milk or preterm formula (that was started on d-3 of life). Although sample sizes were small, both trials were generally of good quality and provided evidence for improved feeding tolerance with parenteral Gln.

More recently, Li et al. [101] have examined the effects of PN supplemented with alanyl-Gln dipeptide for more than 2 weeks in 53 premature infants of LBW. Gln-supplemented infants required fewer d on PN (24.8 versus 30.8 d, *P* < 0.05), had shorter hospital stays (32.1 versus 38.6 d, *P* < 0.05) and fewer episodes of hospital-acquired infections (0.96 versus 1.84 times, *P* < 0.0001) compared to infants who received routine PN. They also regained birth weight sooner (8.1 versus 10.4 d, *P* < 0.05), whereas there were no differences between groups for body weight or head circumference. The results should be interpreted with caution due to limitations in the methodology. It was not clear whether treatment allocation was randomized or whether care givers or assessors were blinded to the intervention. In addition, followup of recruited infants was incomplete. Although 68 infants were enrolled, 15 infants were excluded from the analysis because of insufficient time on PN (<2 weeks) and intention-to-treat analysis was not performed. 

Poindexter et al. [102] performed the largest multicentre trial to determine whether early PN supplemented with Gln reduces the risk of mortality or late onset sepsis in ELBW infants. Within 72 h after birth, 1433 infants were randomly assigned to receive either a standard IV amino acid solution (control) or an isonitrogenous amino acid solution with 20% Gln, whenever they received PN, up to 120 d of age, death, or discharge from hospital. Safety was also assessed in a subset of 141 ELBW infants by measuring plasma concentrations of amino acids and ammonia after infants had received study PN (2.3 ± 1.0 g/kg/d amino acids) for approximately 10 d [103]. While parenteral Gln supplement increased plasma Gln concentrations with no apparent biochemical risk in ELBW infants, Gln did not reduce the incidence of death or late onset sepsis (Gln: 51% versus control: 48%; RR [95% CI]: 1.07 [0.97–1.17]). There were no differences between groups in the number of episodes of late onset sepsis, NEC, d on ventilator, length of hospital stay, d to first and full enteral feeds, feeding intolerance, or growth. Moreover, infants who received Gln required more d of PN support. Although apparently safe in ELBW infants, the authors concluded that parenteral Gln supplementation does not reduce mortality or late onset sepsis, and its routine use cannot be recommended. The lack of significant effect could be explained by a number of factors. Firstly, the primary outcome (death or late onset sepsis) could be influenced by other factors during the clinical course. In addition, as in previous studies [99–101] that used isonitrogenous controls (to ensure the specific effect of Gln), the overall amino acid intake may have been inadequate in the Gln group, as a consequence of the substitution of 20% of the standard amino acids with Gln. Specifically, in order to make the supplements isonitrogenous, amino acids (including essential) were removed from the Gln-containing supplement, which could exacerbate specific amino acid deficiencies (especially if the PN period is prolonged). And although plasma amino acids were similar between groups, comparing plasma amino acid concentrations may not represent a valid marker for nutrient equivalence, since plasma amino acids may not reflect whole body amino acid concentrations or tissue concentrations. Furthermore, because infants in both groups did not receive the targeted amino acid intake of 3.0 g/kg/d until 10 d of age and most had also received small volumes of EN, the delivery of a sufficient dose of Gln may have been inconsistent. Moreover, differences in enteral intake (formula or breast milk) may limit comparability of nutrient intakes between study groups.

More recently, a double blind randomized trial in VLBW infants found no difference in mortality with parenteral Gln supplementation (0.3 g/kg/d) versus control [104]. The trial, however, was not powered to study rare outcomes (such as mortality) or multiple endpoints. Furthermore, no deaths occurred and only 28/30 infants randomized completed the study and thus analysis was not by intention to treat. Interestingly, hepatic function improved as assessed by serum aspartate aminotransferase and direct bilirubin, which both decreased after PN in the Gln-supplemented group (*P* < 0.05). It should be noted, however, that no differences were observed for other measures of hepatic function (bile acid, alanine aminotransferase, total bilirubin, prealbumin, or albumin) or other secondary outcomes (time to full EN, episodes of gastric residuals, total duration of PN, weight gain, head circumference, length of stay, or days on ventilator).

#### 3.1.2. Protein Metabolism

Two small randomized controlled trials in LBW infants examined the effects of Gln-supplemented PN on whole body protein metabolism using primed continuous IV infusions of essential amino acid tracers [80, 105] (Table 1). Des Robert et al. [105] studied 13 LBW neonates on postnatal d-3, while they received exclusive PN that was supplemented with Gln (0.5 g/kg/d) or an isonitrogenous Gln-free amino acid solution for 24 h. Compared to an isonitrogenous amino acid supplement, Gln decreased the rate of plasma Leu appearance, Leu release from protein breakdown (an index of whole body proteolysis; −16%, *P* < 0.05), and rate of Leu oxidation (−35%, *P* < 0.05). There was also, however, a decrease in nonoxidative Leu disposal (an index of whole body protein synthesis; −20%, *P* < 0.05), and, thus, net Leu balance (protein balance) did not differ between groups. Plasma Gln concentrations were higher in Gln versus control, whereas plasma ammonia did not differ. Although parenteral Gln failed to enhance estimates of protein synthesis, Gln may preserve body protein as it suppressed Leu oxidation and protein breakdown in LBW infants. In addition to the small sample size, the failure to enhance protein synthesis may have also been due to insufficient amino acid availability since whole body protein kinetics were assessed on d-4 of life (when amino acid intake was 2 g/kg/d in both groups) before infants received an optimal amino acid intake of 3 g/kg/d [106].

Kalhan et al. [80] examined the effect of 0.6 g/kg/d Gln-supplemented PN for 3–5 d on whole body protein and Gln kinetics in a carefully selected population of 20 clinically stable LBW infants, between d-1 and -2 after birth. Compared to an isonitrogenous control, Gln-supplemented PN resulted in significantly lower rates of appearance of phenylalanine (Phe) and Leu nitrogen and a nonsignificant decrease in the rate of appearance of Leu carbon. Gln also suppressed the endogenous rate of Gln synthesis. There was no significant difference in urea turnover between the 2 groups. The results suggest that parenteral Gln supplementation at 0.6 g/kg/d decreases whole body protein breakdown and Gln *de novo* synthesis in clinically stable LBW infants and may be beneficial in selected populations of LBW infants. The carefully selected population of clinically stable infants limits the application of the results to other groups of premature neonates. Moreover, the use of a higher dose of Gln makes comparisons with other studies difficult.

Interestingly, the same group demonstrated that the suppression of proteolysis and protein oxidation in response to an acute increase in parenteral amino acids (without Gln) was not evident when the amino acid infusion was continued for a prolonged period in both acutely ill [81] and clinically stable LBW infants [82]. The only exception was when amino acids were supplemented with Gln, whereby a prolonged infusion resulted in a sustained inhibition of whole body proteolysis and reduced Gln *de novo* synthesis. Taken together with previous studies [80, 105], Gln supplementation may have a protein-sparing effect in premature infants decreasing whole body protein breakdown and Gln *de novo* synthesis thereby “sparing” the increased amino acids for protein synthesis.

### 3.2. Enteral Glutamine Supplementation in Premature Neonates

#### 3.2.1. Clinical Outcomes

Neu et al. [107] conducted a double-blind randomized trial to test whether enteral Gln supplementation for VLBW infants decreases morbidity (Table 2). Sixty-eight premature neonates were assigned to a Gln-supplemented premature formula or a nonsupplemented standard premature formula between postnatal d-3 to d-30. The Gln supplemented group initially received a dose of 0.08 g/kg/d Gln which was increased to a maximum of 0.31 g/kg/d Gln by d-13. The Gln group had better tolerance to enteral feedings (fewer % of d with no oral intake in Gln: 8.8% versus controls: 23.8%, *P* < 0.01). Episodes of hospital-acquired sepsis were 4/35 and 10/33 in Gln and control group, respectively. Moreover, when controlling for birth weight, the estimated odds of developing sepsis was 3.8 times higher for control versus Gln (95% CI: 1.01–14.18). Analysis of T cell subsets showed a blunting of the rise in HLA-DR+ and CD16/CD56 in the Gln group. There were, however, no significant differences between groups for cases of NEC, growth, or length of stay. Whereas the plasma concentrations of alanine (Ala), glycine (Gly), Ser, threonine (Thr), Phe, and total nonessential amino acids were lower in the Gln-supplemented infants after 2-week supplementation, there were no differences between groups for plasma concentrations of Gln, Glu, or ammonia [108]. The authors speculated that the lower plasma amino acid concentrations in infants fed Gln were the result of enhanced uptake of these amino acids for gluconeogenesis and provide evidence of reduced tissue catabolism. A secondary analysis of the initial trial also provided evidence for decreased hospital costs [109]. While the control used is comparable to routine clinical practice, the study design cannot ensure the specific effect of Gln as the differences between feeding groups might result from higher intakes of nitrogen or energy with Gln supplementation. The authors, however, chose not to use a third group with an isonitrogenous control due to recruitment constraints. Although the trial was small, these initial results provided evidence for better tolerance to enteral feedings and lower sepsis rates in VLBW infants receiving enteral Gln supplementation.

Barbosa et al. [110] conducted a randomized controlled pilot study to evaluate the tolerance and clinical impact of enteral formula supplemented with 0.3 g/kg/d Gln for 5 days versus an equal dose of casein in 9 critically ill infants aged 1–24 months. Although Gln was well tolerated, the study was underpowered to detect differences in septic complications (control: 3/4 versus Gln: 1/5, *P* = 0.10), mortality (control: 2/4 versus Gln: 0/5, *P* = 0.10) or other outcomes (ventilator use, length of stay in intensive care unit (ICU) or in hospital). It was also not reported in the inclusion criteria whether the study population of infants were premature or term.

Vaughn et al. [111] conducted a large multicentre trial to test whether enteral Gln supplement decreases the incidence of hospital-acquired infection and other morbidities in 649 VLBW infants. Within the first 7 d of age, infants were randomly assigned to enteral Gln supplement (0.3 g/kg/d, 3% Gln in sterile water) or placebo (sterile water) given at the same time but separate from feedings for the first 28 d. There were no differences between groups for the primary outcome (nosocomial sepsis between 7 d and 36 weeks postmenstrual age; Gln: 30.9% versus control: 33.7%). However, gastrointestinal dysfunction (2.5 versus 7.5%, *P* < 0.01) and severe neurological sequelae among survivors (Grades 3 and 4 intraventricular hemorrhage and paraventricular leukomalacia; 10.4 versus 15.1%, *P* = 0.08) were less frequent in Gln versus control, respectively. There were no differences in the occurrence of suspected sepsis, pneumonia, urinary tract infection, meningitis, NEC, retinopathy of prematurity, oxygen use at 36 weeks, or mortality. Growth, age, and weight at discharge were also similar. Whereas enteral Gln does not appear to decrease nosocomial sepsis in VLBW infants, the study may have been underpowered to detect a significant difference, as dropout rate was higher than anticipated (i.e., 105 infants exited the study before completion). Also the centre-to-centre variation in this and other multicentre trials [102] may have blunted differences in outcomes (e.g., sepsis), since nutrition and infection control practices may differ among centres and may mask some of the differences that might be apparent in a single facility. While the study provides further evidence to suggest that enteral Gln improves feeding tolerance and may prevent central nervous system (CNS) morbidity, these positive results are based on secondary endpoints and subgroup analyses. Furthermore, the Gln dose was based on birth weight and was not adjusted for interval changes in weight. Therefore, the dose administered may have been inadequate due to rapid growth during the early neonatal period. 

The apparent improved feeding tolerance in VLBW infants receiving enteral Gln in previous studies [107, 111] cannot be explained by enhanced mesenteric blood flow [112]. It seems that, in premature infants without acute illness and tolerating exclusive EN, mesenteric blood flow remains stable after 14 d of age and does not appear to be influenced by enteral Gln.

In contrast to previous reports on Gln-enriched EN in VLBW infants [107, 111], Van Den Berg et al. [113] found no improvement in feeding tolerance, as assessed by the median d to reach full enteral feeds (Gln: 13 d versus control: 13 d; hazard ratio [95% CI]: 1.19 [0.79–1.79]). In this randomized controlled trial, 102 VLBW infants received either enteral Gln supplementation or an isonitrogenous control (Ala) added to breast milk or preterm formula in increasing doses from d-3 to d-30 of life to a maximum dose of 0.3 g/kg/d Gln. There were also no differences between groups for other variables of feeding tolerance (age at which PN was discontinued, d of no enteral feeding), NEC, or growth. However, the Gln-supplemented group had a lower incidence of ≥1 serious infections (sepsis, meningitis, pyelonephritis, pneumonia, and arthritis) compared with the isonitrogenous control group (Gln: 50% versus control: 76%; OR [95% CI]: 0.32 [0.14–0.74]. Other short-term outcomes (patent ductus arteriosus treated with indomethacin or surgical ligation, mechanical ventilation, supplemental oxygen, retinopathy, age at discharge from NICU, age at discharge from hospital, or death) were not significantly different. Gln did not alter plasma concentrations of Gln, Glu, or other amino acids [114]. Although safe at the dose provided, Gln-enriched EN did not improve feeding tolerance or other short-term outcomes in VLBW infants. However, because Gln reduced infectious morbidity, the use of Gln-enriched EN in VLBW infants deserves further consideration. Comparison, however, with other studies is made difficult because of the use of different feeding guidelines for the introduction or withholding enteral feeds. Whereas the choice of isonitrogenous control prevented the removal of amino acids from the Gln supplement in the present study, groups were made isonitrogenous by adding more amino acid (Ala) to the control group. The control group then received additional amino acid/nitrogen, which is not representative of daily practice. However, given the limited sample size, a third comparison group (enteral formula routinely used in an NICU) was not feasible. The beneficial effect of enteral Gln on infection rate could not be explained by an increased number of bifidobacteria or lactobacilli in the intestinal microflora as demonstrated by a secondary analysis in a subset of 86 VLBW infants [115]. Furthermore, Gln did not enhance the postnatal decrease in intestinal permeability, as assessed by the urinary lactulose/mannitol ratio in a subset of 90 VLBW infants. Specifically, supplementation with Gln or isonitrogenous control equally decreased urinary concentrations of lactulose and increased urinary mannitol [116]. More recently, followup of all surviving participants (*n* = 77) revealed that Gln-enriched EN in VLBW infants may lower the incidence of atopic dermatitis (OR [95% CI]: 0.13 [0.02–0.97]) during the first year of life but has no effect on the incidence of bronchial hyperactivity or infectious diseases [117]. Further followup of this cohort of VLBW infants (*n* = 76) at 6 y of age also found a decreased risk of atopic dermatitis (adjusted OR [95% CI]: 0.23 [0.06–0.95]) and gastrointestinal infections (adjusted OR [95% CI]: 0.10 [0.01–0.93]) in the Gln-supplemented group [118]. Although outcomes were assessed by validated questionnaires in these 2 followup studies, parental report of physician diagnosis of disease could be subject to reporting/information bias. Furthermore, the lower incidence of atopic dermatitis and infection rates with Gln were not related to changes in cytokine profiles [119]/responses [120] or to changes in intestinal bacterial species at age 1 y [121]. Finally, the same group studied neurodevelopmental outcome at 2 y corrected age in a subgroup of 72 VLBW infants and found no beneficial effect of Gln-enriched EN during the neonatal period [122].

The effect of Gln supplementation on long-term outcome of VLBW infants has also been reported by Korkmaz et al. [123] who studied the effect of 4-month enteral Gln supplementation on growth. From d-8 through d-120 of life, 69 VLBW infants were assigned to enteral Gln (0.3 g/kg/d) supplement or placebo (sterile water) according to the order of admission to the NICU. Whereas growth parameters did not differ during the first 2 months of life, by the end of the third and fourth month, infants treated with Gln showed higher weight, length, head circumference, mid-upper-arm circumference, and midthigh circumference compared to controls. The authors concluded that long-term enteral Gln in VLBW infants may lead to improvements in growth in a time-dependent manner without any signs of Gln toxicity. Although this was a prospective interventional study, a complete description of masking, blinding, or randomization procedures was not provided. Also, because the placebo (sterile water) was neither isocaloric nor isonitrogenous to the Gln treatment, the enhanced growth could have resulted from the effect of increased amino acid/nitrogen, since early provision of parenteral amino acids (without Gln) has been shown to improve growth parameters in VLBW infants [124].

#### 3.2.2. Protein Metabolism

Darmaun et al. [125] determined the effect of enteral Gln on Leu and Gln metabolism in a subset of 11 VLBW neonates from the larger trial [107] (Table 2). Enteral Gln supplementation provided at low doses (≤0.2 g/kg/d) from d-3 to d-10 of life did not inhibit whole-body protein breakdown in VLBW infants. Leu release from protein breakdown (an index of whole-body protein breakdown) was slightly but not significantly lower in the Gln group versus controls. Plasma Gln concentration, Gln release from protein breakdown, or Gln *de novo* synthesis did not significantly differ between groups. However, there was a trend toward lower rates of Gln *de novo* synthesis in infants receiving Gln supplement. Although the number of patients was small, the failure to detect a significant effect of Gln on its own metabolism or on whole-body protein breakdown could also be due to the different effects of enteral and parenteral Gln supplementation. Importantly, the majority of enteral Gln is used in first pass in premature infants [126]. This is likely a significant factor in the different effects of enteral and parenteral Gln. Moreover, the dose of Gln used in the current study was lower than that (0.5 g/kg/d Gln) previously shown to inhibit proteolysis in LBW infants [105].

Parimi et al. [127] examined the effect of enteral Gln on whole-body Gln and nitrogen kinetics in healthy growing LBW infants during the fasting (3 h after the last meal) and fed state. This study was the only to have 3 groups, where Gln-supplemented group was compared with an isonitrogenous control and enteral formula routinely used in the NICU. Between 10 and 74 d of age, infants were randomly assigned to formula supplemented with Gln (0.6 g/kg/d; *n* = 9), isonitrogenous amounts of Gly (*n* = 9), or unsupplemented formula (*n* = 8) for 5 d. During fasting, the rate of appearance of Phe, Leu carbon, and Leu nitrogen (measures of proteolysis) were not significantly different between groups. Compared with controls, enteral Gln resulted in an increased rate of urea synthesis, no change in Gln rate of appearance, or plasma Gln concentrations. Similar effects were observed with Gly supplement, but the magnitude was less. The authors concluded that enteral Gln does not affect Gln rate of appearance or whole-body protein turnover in a specific group of healthy growing LBW infants, thus, suggesting that Gln is primarily metabolized in the gut (and liver) [126] and is associated with an increased rate of urea synthesis. Alternatively, because, in healthy premature newborns, there is already a high rate of Gln turnover (85% of which is contributed by *de novo *synthesis) [128], this specific population of neonates could have been less sensitive to enteral Gln. This is in contrast to premature neonates with acute illness, whereby catabolic stress may provoke a greater need for exogenous Gln.

In summary, although methodologically sound randomized trials consistently demonstrate safety in VLBW infants [80, 99, 100, 102, 105, 107, 111, 113, 127], parenteral or enteral Gln supplementation does not appear to affect mortality [100, 102, 104, 107, 111, 113], NEC [100, 102, 107, 111, 113], length of stay [99, 100, 102, 104, 107, 111, 113], or growth [100, 102, 104, 107, 111, 113]. Moreover, the results are conflicting for other short-term clinical outcomes such as feeding tolerance [99, 100, 102, 104, 107, 111, 113], serious infections/sepsis [100, 102, 107, 111, 113], ventilator use [99, 102, 104, 107, 113], and severe neurological sequelae [111]. Few data have been reported for the effects of Gln supplementation in VLBW infants on long-term clinical outcomes such as growth at 4 months [123], allergic and infectious morbidity at 1 y [117] and 6 y [118], and neurodevelopmental outcomes at 2 y corrected age [122]. Larger well-controlled studies are needed. A systematic review of 7 randomized controlled trials showed that parenteral or enteral Gln supplementation in premature infants of VLBW does not affect mortality (RR [95% CI]: 0.98 [0.80–1.20]) or other clinical outcomes including invasive infection, NEC, time to achieve full EN, duration of hospital stay, growth, or neurodevelopmental outcomes at 18 months corrected age [129].

In contrast, studies on protein metabolism showed that parenteral Gln may have a protein-sparing effect decreasing whole body proteolysis and Gln *de novo* synthesis in premature infants of LBW [80, 105]. However, the beneficial effects on whole-body protein metabolism have not been reproduced for enteral Gln [125, 127]. Hence, the route of administration (enteral versus parenteral) should be considered in interpreting the effect of Gln on outcome in premature infants [126].

Parenteral and enteral Gln supplementation is apparently safe in premature neonates; however, the lack of any consistent benefit(s) does not support its routine use in this population as a whole. It is possible that any beneficial effects of Gln are limited to specific subgroups of premature infants suffering from acute stress (e.g., NEC, who are perhaps Gln or Arg deficient [88]) whereby increased Gln utilization exceeds the body's synthetic capacity [10]. Future studies are needed to better define the role of Gln in the neonatal period and its mechanism of action. Large prospectively stratified trials are needed to identify the specific subgroups of premature neonates, who may have a greater need for Gln and who may eventually benefit from Gln supplementation.

### 3.3. Glutamine Supplementation in Pediatric Patients with Gastrointestinal Disease

#### 3.3.1. Glutamine Supplementation in Infants with Surgical Gastrointestinal Disease

While several trials have been conducted in VLBW infants, only 2 small double-blinded randomized trials tested whether supplemental Gln might be of benefit in critically ill infants with surgical gastrointestinal disease [130, 131] (Table 3). Duggan et al. [130] compared enteral Gln to an isonitrogenous mix of nonessential amino acids in 20 neonates and infants younger than 12 months receiving PN after gastrointestinal surgery and found no apparent effect on the duration of PN (Gln: 39 d versus control: 21 d, NS) or days to achieve 80% energy requirement by EN (Gln: 24 d versus control: 13 d, NS). Secondary outcomes (energy absorption, clinical infections, or growth) were also not affected by enteral Gln. Albers et al. [131] compared standard PN to isonitrogenous Gln-supplemented PN in 80 newborns and infants (<2 y of age) after major digestive-tract surgery and concluded that parenteral Gln supplementation does not improve intestinal permeability (urinary excretion of lactulose/rhamnose), nitrogen balance, urinary 3-methyl-histidine excretion, or other outcomes (mortality, length of stay in the ICU or hospital, culture-proven sepsis, usage of antibiotics, or ICU resources). Although no adverse effects were identified, both trials as well as meta-analysis for 2 outcomes (in hospital mortality and incidence of invasive infection) [132] do not support the use of parenteral or enteral Gln supplementation in surgical infants with severe gastrointestinal disease until further research proves otherwise.

There are a number of possible explanations for the indeterminate results. Firstly, along with the small sample size, the heterogeneous nature of the infants enrolled may have contributed to the substantial variability in the primary outcome and secondary outcomes and thus limited the power of detecting differences. Whereas the target dose of 0.4 g/kg/d may have allowed Gln supplementation to have an effect, the actual cumulative intake of Gln may not have been adequate to exert its effects. For instance, in the study by Albers et al. [131], tapering of PN (and hence Gln) was allowed to begin only 2 d after 90% of the Gln dose had been achieved, whereas, in that by Duggan et al. [130], inadequate Gln intake may have been due to the gradual advancement of EN and intermittent interruption of feeds required by infants who had undergone intestinal resection. Alternatively, the dose may have been inadequate when administered by the enteral route in this population, since infants may not have absorbed the entire dose of enteral Gln due to their gastrointestinal disease and possible malabsorption [130]. Furthermore, patients fed enterally may require greater protein or amino acids to meet requirements versus those fed parenterally [133]. In addition to the potential effect of route and dose of Gln administration, total nitrogen intake may have been inadequate [131], since nitrogen intake plateaued at 90% of target (<1.5 g/kg/d amino acids), which is lower than the recommended daily allowance for LBW infants or the minimum amount of amino acid needed to prevent protein breakdown [134]. Finally, the isonitrogenous design whereby predetermined amounts of essential and other amino acids were substituted with Gln (as in the studies with VLBW infants) may have obscured potential benefits of Gln supplementation. Large prospectively stratified trials are needed to control for these and other variables that might affect outcome and define precise indications or contraindications for Gln supplementation.

#### 3.3.2. Glutamine Supplementation in Short Bowel Syndrome

Intestinal failure is the inability to maintain nutritional and fluid balance without nutritional support [135]. Short bowel syndrome (SBS) is the result of malabsorption secondary to extensive intestinal resection. The aim in the management of SBS is to enhance intestinal adaptation of the remaining gut in an attempt to achieve intestinal autonomy [135].

Although Gln reduces PN dependence when taken in combination with growth hormone in adults with SBS [136], there is a lack of data on the role of Gln in pediatric patients with this condition. Compared to adult SBS patients, pediatric patients require additional energy and nutrients for growth. Similarly, we have observed increased turnover of protein and Gln in infants compared to adults, regardless of intestinal status [137]. Importantly, previous data from our group also suggests that the infant small intestine plays a prominent role in Gln metabolism and may also be a preferential user of Gln. Specifically, we observed a lower rate of whole-body Gln production and utilization in SBS infants compared to control infants, whereas whole-body protein turnover (Leu appearance rate) was unaltered by intestinal resection [137].

Apart from isolated case reports documenting improvements in weight [138, 139], body composition [139], intestinal permeability [138], stool output [138], and nitrogen retention [139] after supplemental Gln, data are lacking in pediatric patients with SBS (Table 3). In a retrospective review of the medical records of 2 pediatric patients with SBS from neonatal gastrointestinal catastrophes, Ladd et al. [140] observed that late treatment (at ~6 y of age) with growth hormone and concurrent enteral Gln (30 mg/d) supplementation given over long periods improved growth and resulted in PN independence.

Data derived from adults and children with SBS are limited to case series [141, 142] reporting that supplemental Gln (0.6 g/kg/d) over long periods in combination with growth hormone and rehabilitative diet resulted in improved weight [142], plasma proteins [141, 142], intestinal absorptive capacity [141, 142], and weaning from PN [141].

In the above reports, because patients were also receiving other medical therapy, it is not clear whether Gln, growth hormone, diet, or other factors contributed to the favourable outcome. Although no side effects have been reported, data to date are solely hypothesis generating and cannot confirm any benefit of supplemental Gln in promoting intestinal adaptation in pediatric SBS patients. Due to the small number of pediatric patients, lack of control group, and wide patient age range, larger randomized placebo controlled trials of good methodological quality are warranted, especially in children with SBS to delineate whether Gln alone or in combination with other therapies (e.g., growth hormone) is efficacious in intestinal adaptation. This highlights the need for further studies to define specific selection criteria with respect to patient age (e.g., infants or children). Consideration should also be given to the timing of treatment initiation, that is, the duration between intestinal resection and start of treatment (e.g., early versus late treatment).

#### 3.3.3. Enteral Glutamine in Children with Crohn's Disease

Children with active Crohn's disease undergo catabolic stress, demonstrating increased rates of whole-body protein turnover [143]. Whereas animal models of IBD have indicated potentially beneficial effects of supplemental Gln [144–146], clinical studies of Gln in inflammatory bowel disease (IBD) have been less encouraging [147–149].

Akobeng et al. published a series of reports [148, 150–152] (Table 3) from a group of 18 children (aged 6.8–15.7 y) with active Crohn's disease who participated in a double-blind randomized controlled trial comparing the efficacy of 4 weeks exclusive enteral Gln-enriched polymeric diet (42% of amino acids as Gln) with a standard polymeric diet (4% of amino acids as Gln) that was isocaloric and isonitrogenous [148]. The initial trial found no evidence that Gln-enriched polymeric EN is of any benefit over standard polymeric EN in the treatment of children with active Crohn's disease. After 4 weeks of exclusive EN, there were no differences in remission rates (4/9 in Gln versus 5/9 in control), changes in platelet count, orosomucoid level, or weight. Gln, however, was less effective in improving indices of disease activity (pediatric Crohn's disease activity index (PCDAI)) compared to standard diet.

Secondary analyses in a subset of children from the initial trial also showed that Gln was equivalent to isonitrogenous standard in attenuating increased intestinal permeability [150] or improving plasma antioxidant status [152], possible factors in the pathogenesis of Crohn's disease. Furthermore, Gln-enriched EN did not lead to any improvement in the reduced IGF1 concentrations described in children with Crohn's disease and was actually less effective than standard isonitrogenous EN in improving impaired growth in these children [151].

The inconclusive results could be explained by baseline differences in the PCDAI, which (although adjusted for in the analysis) may have contributed to the greater improvement in PCDAI with the standard diet. It is also possible that such a high concentration of Gln used in the present study may have exceeded the optimal concentration of Gln, that is, beneficial and may actually be harmful in IBD [153]. Gln might have caused excessive priming of immune cells [154], which in turn may promote inflammatory activity. This highlights the need for dose-finding studies in this population. Alternatively, substitution of 42% of amino acids with Gln may have led to an imbalance in the amino acid profile.

Results are indeterminate from this one small trial [148]. Moreover, 2 children (both from the Gln group) were withdrawn from the trial, because of nontolerance of the diet. Mechanistic data are needed to better understand how Gln interacts with disease pathogenesis and to determine whether Gln is in fact contraindicated or beneficial in children with Crohn's disease, and if so at which doses. Fundamental research is needed before any further randomized controlled trials can be conducted with larger numbers of these children.

### 3.4. Enteral Glutamine in Children with Diarrheal Disease/Malnutrition

#### 3.4.1. Enteral Glutamine Supplementation in Infants and Children with Diarrhea

In animal models (rat and rabbit) of secretory diarrhea induced by cholera toxin, Gln-based oral rehydration solution (ORS) was more effective in inducing the absorption of electrolytes and water versus glucose-based ORS [155, 156]. Similarly, in experimental bacterial diarrhea using the Escherichia coli model, Gln-containing ORS corrected plasma volume and prevented significant weight loss in diarrheic calves [157]. Likewise, infected rabbits also show enhanced intestinal sodium absorption with enteral Gln treatment [158]. In children, amino acids (e.g., Gln) may have facilitating effects on fructose and sorbitol absorption [159].

Three double-blind randomized controlled trials have tested the efficacy of oral Gln in the treatment of diarrheal disease in infants and children [160–162] (Table 4). Ribeiro Jr. et al. [160] studied 118 infants (1–12 months of age) with acute noncholera diarrhea and dehydration and reported that Gln-based ORS did not provide additional benefit over the standard World Health Organization (WHO)-ORS with respect to diarrheal stool output, duration of diarrhea, or volume of ORS needed to achieve/maintain hydration. This initial trial was generally of good methodological quality; however, the addition of Gln to the standard WHO-ORS led to a higher osmolality versus standard, and osmolality could affect diarrheal symptoms. More recently, Gutierrez et al. [162] have compensated for the hyperosmolality by testing an ORS in which glucose was replaced with Gln in 147 dehydrated children (aged 1–60 mo) with acute diarrhea compared to standard glucose-based WHO-ORS. Although solutions were of similar osmolality, glucose-free Gln-based ORS did not appear to reduce stool output, volume of ORS or time required for rehydration, urine output, or vomiting. The indeterminate results of both trials could be explained by subjects being only mildly to moderately dehydrated, since ORS is more effective when given to dehydrated patients [160, 161]. Furthermore, the early feeding just after rehydration may have blunted the effects of Gln [160]. Finally, Gln may have a lesser effect on intestinal absorption of water and sodium in noncholera diarrhea compared to when the secretory component of the diarrheal episode is more severe (i.e., when there is a relatively larger secretion of sodium and chloride) [155, 156]. For instance, in pig rotavirus enteritis, Gln stimulates jejunal sodium and chloride absorption [163].

Yalçin et al. [161] administered oral Gln (0.3 g/kg/d) as capsules for 7 d in 128 otherwise healthy infants aged 6 to 24 months with acute diarrhea. Compared to cornstarch placebo, Gln reduced the duration of diarrhea (Gln: 3.40 ± 1.96 d versus placebo: 4.57 ± 2.48 d, *P* < 0.01) for the entire cohort and among infants aged ≥12 mo, but not for infants <12 mo. The frequency of persistent diarrhea (Gln: 2/63 versus placebo: 6/65) or vomiting (Gln: 24/63 versus placebo: 32/65) was not significantly different. Serum interleukin-8 was significantly less after 7-d treatment in both groups with no difference between groups, whereas salivary immunoglobulin A showed no change. No differences in leukocyte counts, lymphocyte counts, or lymphocyte subpopulations were observed between Gln-supplemented and placebo groups [164]. Furthermore, during the 3 months after treatment, mean weight gain and incidence of infections were similar in both groups. Thus, the reduction in diarrheal duration by Gln seems to result from its local beneficial effect on the gastrointestinal mucosa rather than a modulation of the immune response. Alternatively, the lack of immunomodulatory effect may have been partly attributed to patient selection of children with mild to moderate diarrhea, who may have responded differently to those with persistent or secretory diarrhea. It is also possible that some infants received Gln from breast milk and, thus, were not in fact truly Gln deficient, especially infants <12 mo (which could explain the lack of effect in this subgroup). The results, however, should be taken with caution as the primary outcome was a subjective measure based on patient self-report (i.e., clinical recovery as described by the mother and noted in an observation chart given to the mother). Also the small number of children may have limited the statistical power to detect changes in complications that are less frequent (e.g., frequency of persistent diarrhea).

#### 3.4.2. Enteral Glutamine Supplementation in Malnourished Infants and Children

In the malnourished growing rat, Gln-enriched diet exerts trophic effects on the jejunal mucosa leading to improvements in body weight gain [165]. Similarly, feeding Gln to weaned piglets alleviates growth depression resulting from Escherichia coli challenge, via the maintenance of intestinal morphology and function [166]. Three double-blind randomized controlled trials assessed intestinal permeability using the urinary excretion of lactulose/mannitol ratio to examine whether oral Gln could improve intestinal barrier function in malnourished children and thereby enhance growth [167–169] (Table 4). Lima et al. [168] studied 80 malnourished hospitalized children aged 2 to 60 months to compare enteral formula supplemented with Gln (16.2 g/d) versus formula supplemented with isomolar Gly (8.3 g/d) or formula alone for 10 d. There was a decrease in the lactulose/mannitol ratio after 10 d Gln supplementation, which was not observed after Gly nor standard nonsupplemented formula. However, the % urinary excretions of lactulose or mannitol were not affected. Furthermore, the mean duration of diarrhea, weight gain, or weight-for-age z-scores did not differ among groups; however, there was a trend for weight to increase in children supplemented with Gln or Gly, compared to nonsupplemented diet. Although the study compared Gln-supplemented formula to Gly-supplemented and a nonsupplemented formula, allocation to the nonsupplemented group was not randomized, and these children were studied 2 y prior to the randomized trial. Moreover, in the supplemented groups, the Gln and Gly consumed per d contained different amounts of nitrogen, 3.1 g and 1.6 g, respectively. Thus, the effect of Gln (independent of the amount of nitrogen) cannot be confirmed.

A subsequent trial by the same group [169] also demonstrated improved barrier function, measured by decreased % urinary lactulose excretion in 107 children (7.9 to 82.2 months of age) administered oral alanyl-Gln (24 g/d) for 10 d, but not with isonitrogenous Gly (25 g/d). However, alanyl-Gln also led to a reduction in % urinary excretion of mannitol after 10 d, resulting in no change in the lactulose/mannitol ratio and no difference between groups. Interestingly, alanyl-Gln led to improvements in cumulative change over 120 d of weight-for-age and weight-for-height z-scores (but not height for age). Although it appears that oral alanyl-Gln supplement may improve intestinal barrier function and reduce wasting in malnourished children, absorptive epithelium is also reduced as determined by a decrease in mannitol recovery. The decreased urinary excretion of mannitol could be related to the high dosage used in this study (~1.79 g Gln/kg/d). This study [169] and the previous [168], however, may have been underpowered to detect an improvement in diarrheal disease morbidity, which was a secondary parameter (not used in the sample size calculation). Moreover, a longer supplemental period was likely necessary to demonstrate significant effects. Finally, whereas this subsequent trial used an isonitrogenous comparison group, the potential intestinal trophic effect of Gly limits its use as a control for Gln [6, 170]. This is also supported by experimental data showing that ORS containing Gln is equivalent to Gly in enhancing mucosal regeneration or maintaining hydration status in calves with viral-induced diarrhea [171].

Williams et al. [167] used a longer supplemental period of oral Gln (0.5 g/kg/d) in 93 malnourished Gambian infants (aged 4 to 11 months) during the 5-6 month rainy season but failed to demonstrate improvements in growth, intestinal barrier function, or plasma markers of immunostimulation compared to an isonitrogenous control. Growth was poor and was reflected by reductions in weight-for-age, height-for-age z-scores, and mid-upper-arm circumference during the 5-month supplemental period with no difference between groups. Furthermore, supplementation with either Gln or isonitrogenous amino acids did not improve lactulose/mannitol ratios or % recovery of lactulose or mannitol. Plasma immunoglobulin (Ig) concentrations (IgA and IgG) increased during the study, whereas IgM, albumin, antichymotrypsin, and C-reactive protein showed no change. Moreover, morbidity or plasma markers of immunostimulation were not affected by Gln. The lack of significant effect could have been due to the possible trophic effects on the intestine of nitrogen or amino acids (e.g., Gly, Glu, and Cys) in the control group [6, 170], which consisted of a mix of nonessential amino acids. Alternatively, the constant daily exposure of these infants to pathogens or the possible influence of underlying diseases may have prevented any improvement with Gln. Finally, infants may not have been truly Gln deficient as some received Gln from breast milk or other dietary sources. However, food intake data (breastfeeding in particular) was lacking.

Although apparently safe and well tolerated, results from double-blind randomized controlled trials are conflicting regarding the efficacy of oral Gln in the treatment of diarrheal disease [160–162, 168] or in enhancing intestinal barrier function [167–169] or growth [167–169] in children suffering from diarrhea or malnutrition. Furthermore, any beneficial effect of oral Gln does not seem to result from a modulation of the immune response [161, 167].

Large randomized controlled trials are needed with adequate power, treatment durations, and objective outcome measures. Also because specific subgroups of infants and children may or may not respond to supplemental Gln, patient selection criteria should be clearly defined, particularly with respect to diarrheal disease severity (e.g., secretory diarrhea), hydration status (e.g., severely dehydrated), or age (e.g., infants ≥ 12 mo). The choice of control (e.g., isonitrogenous) is also an important issue in the design of these studies due to a potential intestinal trophic effect of other amino acids, such as Gly [6, 170]. Finally, consideration should be given to the possible influence of factors, such as underlying disease, complications, or systemic infections as well as food intake (particularly breastfeeding in infants).

### 3.5. Glutamine Supplementation in Pediatric Oncology Patients

Some studies in adult cancer patients indicate beneficial effects of Gln supplementation after chemotherapy-radiotherapy or bone marrow transplant (BMT) [172]. In the rat model of methotrexate-induced injury, specific nutrients (such as Gln) and growth factors provide protection against gastrointestinal damage and weight loss [173]. However, data are limited on the effects of Gln supplementation in pediatric oncology patients. An initial case report in a child receiving chemotherapy showed decreased costs and good tolerance with Gln-supplemented tube feedings [174]. Subsequently, Pietsch et al. [175] assessed the feasibility of administering Gln-enriched nasogastric enteral feedings for ~12.7 d in 17 children (aged 2–19 y) receiving intensive chemotherapy or BMT and reported minimal complications and reduced hospital costs versus PN (Table 5). Ward et al. [176] conducted a pharmacokinetic dose-finding study in 13 pediatric oncology patients (aged 3–18 y) undergoing chemotherapy and concluded that oral Gln at a single dose up to 0.65 g/kg was well tolerated and safe to use in a clinical study in pediatric oncology patients. However, it was not feasible to assess the dose-limiting toxicity at higher doses. Apart from these preliminary studies and a case-control study that examined parenteral Gln [177], all other trials in children undergoing treatment for cancer tested oral Gln at a daily dose of 4 g/m^2^ body surface area (BSA) [178–181] (Table 5).

#### 3.5.1. Glutamine Supplementation on Oral Mucositis in Children Undergoing Chemotherapy or Bone Marrow Transplant/Hematopoietic Stem Cell Transplant

Painful oral mucositis is a common complication after chemotherapy or BMT/hematopoietic stem cell transplant (HSCT) resulting in reduced oral intake and frequently requiring PN. In a small case-control study, Kuskonmaz et al. [177] matched 21 pediatric cases (mean age 8.3 ± 5.2 y), who underwent allogeneic HSCT and received parenteral Gln supplementation (0.4 g/kg/d) to a group of 20 control children (comparable with respect to donor type, diagnosis, and age) and observed a trend toward reduced incidence of mucositis (29 versus 55%, *P* = 0.118), sinusoidal obstruction syndrome (10 versus 35%, *P* = 0.067), and drug-related toxicity (14 versus 40%, *P* = 0.085) (Table 5). Gln supplementation was also associated with reduced duration of fever (5.7 versus 12.0 d, *P* = 0.021); however, no differences in total parenteral nutrition (TPN) use, neutrophil engraftment, Graft Versus Host Disease, length of stay in hospital, infections, or mortality were observed.

Three double-blind randomized trials tested the efficacy of oral Gln compared to Gly placebo in reducing oral mucositis [178–180] (Table 5). Anderson et al. [179] conducted a crossover study whereby 24 patients (16 children and 8 adults) were randomly assigned to oral Gln over 2 courses of chemotherapy and Gly placebo over another 2 courses (or the reverse order). Supplementation was then continued for another 14 d after chemotherapy. Although the duration and severity of mucositis was reduced in the chemotherapy courses in which Gln was provided (4.5 d less with Gln versus placebo, *P* < 0.001), results were based on patient self-report by means of a calendar completed by the patients on the duration and severity of mucositis associated with each chemotherapy course. Also in addition to the small sample size, followup of recruited patients was incomplete, as only 13 patients actually completed at least 2 courses of identical chemotherapy (10 children aged 4–17 y and 3 adults). Hence additional studies with larger numbers were needed.

These issues were addressed in a larger cohort of 193 patients (including 72 children) undergoing BMT, whereby the same group assessed mucositis by both self-report and by opiate use [180]. Patients were prospectively stratified by type of transplant (autologous, matched sibling donor or unrelated donor) and randomized to receive either oral Gln or Gly placebo during preparative chemotherapy and radiation until 28 d after BMT. In autologous BMT patients, Gln reduced the severity of oral mucositis assessed by self-report and by duration of opiate use (Gln: 5.0 ± 6.2 d versus control: 10.3 ± 9.8 d, *P* < 0.01). By contrast, in matched sibling BMT patients, Gln had no effect on self-report and actually increased the duration of opiate use (Gln: 23.2 ± 5.7 d versus control: 16.3 ± 8.3 d, *P* < 0.01), whereas % survival at d-28 was increased by Gln in allogeneic patients. Although other outcomes (TPN use, rate of relapse or progression of malignancy, antibiotic use, graft versus host disease, d of hospitalization, infections, or survival at d-100) were not affected, the results suggest that oral Gln is safe and effective in decreasing the severity and duration of mucositis in autologous but not allogeneic BMT patients. The study population was heterogeneous in terms of age (range: 1–62 years); subgroup analysis, however, of the 72 child participants was not reported.

Aquino et al. [178] further demonstrated the safety and benefit of oral Gln in reducing the severity of mucositis in 120 children undergoing HSCT. Compared to Gly placebo, the Gln group showed a trend toward a reduction in the mean mucositis score (Gln: 3.0 ± 0.3 versus Gly: 3.9 ± 0.4, *P* = 0.07) as well as a reduction in the number of d of IV narcotics use (12.1 ± 1.5 d versus Gly: 19.3 ± 2.8 d, *P* < 0.05) and TPN (17.3 ± 1.7 d versus Gly: 27.3 ± 3.6 d, *P* = 0.01). There were no differences between groups for the number of episodes of bacteremia, total number of hospital d, or mortality. Although consideration should be given to include oral Gln supplementation as part of the supportive care of HSCT, the primary endpoint (mean mucositis score) was subjectively measured by a clinical scoring scale, which is a limitation of this and other studies that assessed mucositis severity. Furthermore, the study may have been underpowered to detect a significant difference, since only 100 mucositis scoring sheets were available for analysis, whereas the estimated sample size was 120 patients. Finally the lower incidence of herpes simplex virus-positive children (a known risk factor for the development of mucositis [178]) in the Gln group at baseline may have confounded the results.

#### 3.5.2. Glutamine on Immune Function: Implications for Children with Solid Tumors and Acute Lymphoblastic Leukaemia

In patients with solid tumors and lowered lymphocyte proliferative response, Gln treatment may improve immune function. Köhler et al. [182] studied the influence of Gln and glycyl-Gln on the proliferative response of lymphocytes isolated from 21 children (aged 1–17 y) with solid tumors, before and after chemotherapy and observed no difference in the *in vitro* lymphocyte proliferation before or after chemotherapy. Although these *in vitro* data do not support the routine use of Gln supplementation to enhance lymphocyte function in children with solid tumors, specific subgroups of tumors showed trends that differed from the overall findings, but statistical power was inadequate to prove any differences.

An *in vivo* study by Okur et al. [181] examined 21 children (aged 1–17 y) with solid tumors to compare the effects of oral Gln supplementation during a 5-d chemotherapy course versus another course without Gln and showed that the immune-enhancing effects of Gln could involve not only lymphocytes but also the complement pathway (Table 5). Moreover, Gln supplementation was associated with significant improvements in some nutritional parameters and reduced stomatitis severity and antibiotic necessity, suggesting that oral Gln could be considered in children with solid tumors receiving chemotherapy.

A recent report in mice has suggested that supplementation with alanyl-Gln could support the peripheral immune system and cell-mediated immunity during asparaginase chemotherapy [183]. This has implications for children since asparaginase which is used in the treatment of acute lymphoblastic leukaemia (ALL) (the most common childhood cancer) also depletes plasma asparagine and Gln [184, 185] resulting in immunosuppression. Moreover, low plasma concentrations of gluconeogenic amino acids, especially Gln and Ala have been reported during chemotherapy-related hypoglycaemia in ALL children [186], which is in contrast to the increased Gln concentrations observed in the cerebral spinal fluid of children with ALL undergoing chemotherapy or with CNS disease [187]. Future animal research is needed to test the safety and efficacy of Gln during asparaginase chemotherapy before trials in children with ALL.

Results from 3 randomized trials suggest that oral Gln supplementation is safe and may reduce the severity and duration of oral mucositis in patients undergoing treatment for cancer, including chemotherapy, autologous BMT, and HSCT [178–180]. Although trials were methodologically sound, sample sizes were small, and 2 trials included both pediatric and adult patients. Moreover, outcome (mucositis severity) was assessed subjectively by the patient rather than objectively by an independent observer.

The data from nonrandomized preliminary clinical studies demonstrate safety [176] and suggest that supplemental Gln may lead to reduced hospital costs in children receiving chemotherapy or BMT [175], reduced fever duration in children undergoing HSCT [177], and improvements in nutritional and immunological parameters in children with solid tumors receiving chemotherapy [181]. However, *in vitro* data do not support the routine use of Gln supplementation to enhance lymphocyte function in children with solid tumors [182].

Due to the lack of data in children, additional randomized controlled trials are needed with adequate numbers of pediatric oncology patients to confirm any benefit of supplemental Gln. Future trials should define the patient populations for which Gln may or may not be of benefit, by using clearly defined selection criteria with respect to age (e.g., pediatric versus adult), specific cancer treatments (e.g., autologous versus allogeneic BMT), or subgroups of malignancies (e.g., specific subgroups of solid tumors) and evaluate efficacy using objective outcome measures that are both valid and reliable.

### 3.6. Glutamine in Pediatric Patients with Severe Burns/Trauma

In situations of abnormal muscle protein metabolism (e.g., severe burns or trauma), endogenous Gln production could be impaired [188]. Gore and Jahoor [188] observed decreased arterial Gln concentrations and decreased rates of Gln turnover, in 5 severely burned children compared to control children, whereas net efflux of Gln did not differ. Thus, suggesting that reduced plasma Gln, observed in burned children, results from a deficiency in peripheral Gln production and increased central consumption. This supports the notion that Gln supplementation may be needed in pediatric burn patients because of an inadequate skeletal muscle response.

The reduced plasma Gln concentrations observed after major burns could also in part contribute to the immunosuppression, that is, seen in these patients. An *in vitro* study by Ogle et al. [189] isolated neutrophils from 12 pediatric burn patients (aged 4–18 y) at various postburn times to determine their ability to kill Staphylococcus aureus in the presence or absence of Gln (20 mmol/L) and compared this with normal subjects. At all but 2 postburn times, Gln improved the observed abnormal neutrophil bactericidal function, restoring it to normal levels. Although Gln had no effect on C3b receptors (CR1) or on phagocytosis, the restoration of the impaired bactericidal function shown in this *in vitro* study provided additional evidence for Gln supplementation in the diets of pediatric burn patients.

There exists, however, limited clinical data demonstrating efficacy of supplemental Gln in burn and other trauma patients (Table 6). Sheridan et al. [190] conducted the only randomized double-blind trial of supplemental Gln using a crossover design in 9 pediatric burn patients (aged 1.3–15.8 y) who received in a random order: 48 h of standard enteral feedings (control phase) and 48 h of enteral feedings with Gln (0.6 g/kg/d) replacing 20% of essential and nonessential amino acids. Whole-body protein kinetics, determined in the fed state at the end of each study phase, revealed that Leu flux and Leu oxidation were lower during Gln supplemental period versus control period. However, no significant differences were observed in net balance of Leu accretion into protein, nitrogen balance, or plasma Gln concentrations. The lack of beneficial effect on protein accretion could have resulted from a number of reasons. Firstly, the use of an isonitrogenous control tests the specificity of Gln but also results in a reduction in both essential and nonessential amino acids in the Gln diet, which may have counterbalanced any beneficial effect of Gln. Furthermore, the risk of type II error cannot be ruled out, since only 7 out of the 9 children enrolled actually completed the study. Finally, the Gln feeding period may have been too short to replenish the depleted Gln pools in stressed pediatric burn patients. Hence, a longer supplemental period may be required to restore plasma Gln concentrations and stimulate protein accretion in this population.

Microdialysis and magnetic resonance spectroscopy observations in children indicate that the Gln/Glu balance in the injured brain may play a significant role in the pathophysiology of traumatic head injury in children [191–193]. Moreover, higher brain Glu/Gln ratio correlates with increased tissue damaging procedures in asphyxiated term neonates [194]. The efficacy of supplemental Gln in severe trauma was tested in 2 small trials that included both pediatric and adult patients [195, 196] (Table 6). Yang and Xu [196] compared Gln-Ala dipeptide versus routine nutritional therapy in 46 patients aged 7–68 y with severe traumatic brain injury. Chuntrasakul et al. [195] compared immuno-nutrient formula enriched with Gln versus standard EN in 36 patients aged 15–60 y with severe burns/trauma. Results were inconclusive since studies were underpowered to detect statistically significant differences, particularly for nonspecific, multifactorial outcomes, such as length of stay and mortality. Also the clinical heterogeneity (wide age range) of subjects included limits application to children. Apart from methodological issues (e.g., incomplete description of blinding and randomization procedures), the study by Chuntrasakul et al. [195] used a commercial product that combined Gln with added Arg and omega-3 fatty acids. As such, it is difficult to assess the effects of Gln apart from the effects of other nutrients. Furthermore, the actual intake of Gln was not significantly greater in the study group versus control (≤0.15 versus ≤0.07 g/kg/d, resp.). Thus, any effect of Gln may have been blunted.

In summary, although *in vitro* data suggest beneficial effects of supplemental Gln in pediatric burn patients [189], clinical data are less encouraging [190]. Short-term enteral Gln is apparently well tolerated but does not result in an immediate whole body protein gain in pediatric burn patients [190]. Moreover, studies showing benefits of supplemental Gln in pediatric and adult patients with severe trauma are inconclusive, and methodological problems have been noted [195, 196]. Larger well-designed studies are needed, especially in children to test any clinical benefit. Protein kinetic studies should consider the duration of administration, for example, testing enteral Gln administered over longer periods on protein metabolism as well as on its own metabolism to determine whether Gln's protein-sparing effect also applies to burned children. Such studies might also consider the route of administration, as no study has yet tested the efficacy of parenteral Gln on protein metabolism or on clinical outcome in pediatric burn patients.

### 3.7. Enteral Glutamine Supplementation in Chronic Diseases of Childhood

#### 3.7.1. Enteral Glutamine Supplementation in Children with Duchenne Muscular Dystrophy

Duchenne muscular dystrophy (DMD) is an X-linked disease caused by a defect in the gene encoding for dystrophin (a cytoskeletal protein) [197]. The absence of dystrophin expression is associated with a progressive and severe loss of muscle mass and function. Studies on protein metabolism suggest that muscle wasting in DMD could result from a reduction in muscle protein synthesis or an increase in protein degradation or both [198–200]. Because skeletal muscle is the body's main producer and exporter of Gln and muscle mass is drastically reduced in DMD, the need for Gln may be increased in persons who have this disease. Furthermore, in DMD as in other protein-wasting conditions, the intramuscular Gln concentration is low [201, 202]. And Sharma et al. [202] suggested that this may be an underlying reason for muscle wasting in DMD. Additionally, we have demonstrated that compared to normal boys, the dramatic muscle mass loss observed in DMD boys aged 10 y was associated with a decrease in whole-body Gln availability in the postabsorptive state, resulting from a decrease in Gln *de novo* synthesis [203]. Thus, in DMD as in other catabolic stress conditions, Gln might be considered a conditionally essential amino acid. 

We conducted 2 separate studies using stable isotope methodology [204, 205] (Table 7), to test the effect of oral Gln on whole-body protein and Gln metabolism in DMD children during the postabsorptive state. In the initial study [204], Leu and Gln kinetics were measured in 6 DMD boys aged 8–13 y on 2 consecutive days while children received a 5-h oral administration of flavoured water (Kool-Aid) on the first study day followed by Gln (0.6 g/kg) dissolved in the same flavoured water on study day-2. During Gln administration, Leu release from protein breakdown and Leu oxidation rate both decreased by 8% and 35%, respectively (*P* < 0.01), resulting in no change in nonoxidative Leu disposal (an index of protein synthesis). Whereas, whole body Gln exchange in the plasma doubled (*P* < 0.01), Gln from protein degradation and Gln *de novo* synthesis both decreased during oral Gln administration. These preliminary data suggested that acute oral Gln might have an acute protein-sparing effect in children with DMD resulting from a decrease in whole-body protein degradation and Gln *de novo* synthesis. However, results should be taken with caution due to the small sample size. Additionally, the order of treatment allocation was not randomized, and participants and assessors were not blinded to treatment. Moreover, the specificity of Gln's effect on protein metabolism could not be tested as measurements were not performed using an isonitrogenous control group as well.

We addressed these shortcomings more recently by conducting a double-blind randomized controlled trial [205] in 26 DMD boys (aged 7–15 y) to test whether the acute protein-sparing effect of Gln persisted when oral Gln (0.5 g/kg/d) was given for 10 d and whether the effect was specific to Gln, by comparing the Gln-supplemented group to an isonitrogenous control (amino acid mixture). Whereas plasma Gln concentrations were not altered this time (since kinetic studies were performed 24 h after Gln or amino acid administration), the 9% (*P* < 0.05) decrease in Leu release from protein breakdown persisted after 10 d Gln supplementation and endogenous Gln from protein degradation also decreased. Similar effects were observed after 10 d amino acid supplementation; however, the magnitude of the decrease in whole-body proteolysis was less (−4%, *P* < 0.05). There were no significant effects on other estimates of Leu and Gln turnover or on body composition (fat-free mass, % fat mass, muscle mass, and weight) after 10 d supplementation in either group. The lack of significant difference between Gln and isonitrogenous control could be explained by the variability in disease progression among the study population, since Gln treatment in DMD may have different effects depending on the stage of the disease process [206]. It is also possible that a higher dose or longer treatment duration may be necessary to demonstrate the specific effect of Gln, separate from its role of providing nitrogen. This highlights the need for dose and time course data on Gln administration in DMD. Alternatively, the route of administration (enteral versus parenteral) could partly explain the lack of significant difference, since Gln's protein-sparing effect may be less dramatic when it is given enterally [125, 127] as opposed to parenterally [80, 105], as demonstrated in studies on protein metabolism in premature infants of LBW [126]. 

Based on experimental data showing that Gln improved performance in the *mdx* mouse model of DMD [207], Escolar et al. [206] conducted a randomized double-blind placebo-controlled multicentre study to test the efficacy and safety of 6-month oral Gln (0.6 g/kg/d) in 35 ambulant steroid naïve boys with DMD aged 4–10 y (Table 7). Whereas there were no significant differences in the primary outcome (manual muscle testing score) or on quantitative measurements of muscle strength, subgroup analysis showed that in younger boys (<7 y) the Gln group had significantly less deterioration over 6 months in timed functional tests versus placebo. Although there was a trend toward less deterioration in quantitative and functional measures of muscle strength with Gln treatment over 6 months, the effect did not reach significance for the cohort as a whole. The inability to detect a significant difference in the primary outcome could be explained by the unexpected lack of strength deterioration (measured by manual muscle testing) in the placebo group over the 6-month trial. Thus, the study may have been underpowered since power calculations were based on previous natural history data of DMD [208]. The significant age-related results must be interpreted with caution as they were based on an unplanned subgroup analyses in a small group of patients. Larger trials incorporating *a priori* age stratification are required to test the disease-modifying effect of long-term Gln supplementation in DMD.

Our group has recently completed a multicentre randomized controlled crossover trial in 30 prepubertal DMD boys aged 2–10 y to test whether 4-month oral Gln can slow the progressive loss in muscle mass and function that occurs in these children [209]. Subjects received 4-month Gln separated by a 1-month washout, followed by 4-month placebo (maltodextrin) or vice versa. The order of treatment allocation was randomized. Overall, there was no apparent functional benefit as tested by comparing Gln versus placebo on change in walking speed at 4 months (primary outcome) or in secondary measures of muscle function (2-minute walk test, work, and power). We observed no differences in muscle mass (urinary creatinine), markers of protein breakdown (urinary 3-methyl-histidine/creatinine), or serum creatine phosphokinase in the Gln group compared with placebo, except for a blunted increase in fat free mass in the Gln group which led to a greater increase in fat-mass percentage. Our findings that functional measures did not deteriorate during the 4-month placebo phase or over the course of the 9-month trial were not as expected. Based on natural history data [208], the trial was powered to detect a 10% difference in walking speed after 4-month Gln compared to placebo. However, we did not consider the greater placebo effect reported in children [210] which could have narrowed the expected effect size of Gln treatment. Interestingly, subgroup analysis revealed a significant effect of Gln treatment on functional measures in boys taking corticosteroids (*P* < 0.05). Specifically, boys taking corticosteroids showed a significant decline in walking speed during the placebo phase (−40%, *P* < 0.05), whereas walking speed remained stable when corticosteroid-treated boys received Gln treatment for 4 months. Although the findings must be interpreted with caution, because they derive from an unplanned analysis in a small subgroup of boys (*n* = 5), they might suggest a rationale for Gln supplementation in conjunction with corticosteroid therapy which needs investigation.

In summary, studies on protein metabolism in DMD children suggest a protein-sparing effect of Gln, resulting from a decrease in whole-body proteolysis [204, 205]. Moreover, we have identified a potential antioxidant protective mechanism for Gln's antiproteolytic effect in dystrophic muscle of young *mdx* mice [35]. Although safe, supplemental Gln does not appear to improve muscle strength [206] or function in DMD children [209]. Better targeting of specific subgroups (younger DMD children or DMD children under stress, e.g., corticosteroid treatment) is necessary to fully evaluate the presence (or absence) of benefits. Fundamental research is also needed to elucidate potential mechanisms whereby therapies such as Gln target events downstream of the genetic defect in the progressive dystrophic pathology.

#### 3.7.2. Enteral Glutamine Supplementation in Children with Sickle Cell Anemia

Children with sickle cell anemia often have decreased height and weight and reduced muscle mass when compared with healthy children [211, 212]. This may be partly due to increased metabolism, since prepubertal children with sickle cell disease have been shown to use approximately 19% more energy, 58% more protein, and 47% more Gln compared with age-, sex-, and race-matched controls [213], even in the absence of sickling, vaso-occlusive disease or intercurrent illness, suggesting that sickle cell children may have greater protein and energy needs than healthy children, and this could lead to impaired growth. The increased protein and energy needs could be due in part to the increased erythrocyte production as a result of the chronic hemolysis and anemia that leads to increased cardiac output [213]. In adult sickle cell disease patients, oral Gln has been shown to improve NAD redox potential of sickle red blood cells and may result in reduced oxidative stress susceptibility [214]. Moreover, low erythrocyte Gln may contribute to alterations in the erythrocyte redox environment and may play a role in hemolysis and pulmonary hypertension in patients with sickle cell disease [215]. As well, reduced concentrations of plasma Gln have been reported in children with sickle cell disease [216], malaria [217, 218], and *β*-Thalassemia [219], which may be related to the growth impairment in height and weight also observed in these children.

Whereas previous data suggest positive physiological effects of Gln in sickle cell disease [214, 220, 221], Williams et al. [222] were the only group to have examined the role of Gln in children who have this disease (Table 7). They studied 27 children with sickle cell anemia (aged 5.2–17.9 y) and observed that oral Gln (0.6 g/kg/d) administration for 24 weeks was associated with a decrease in resting energy expenditure and increased body mass index, % fat mass, muscle strength, and plasma concentrations of Gln compared to baseline. Subgroup analysis in underweight children showed an even greater decrease in resting energy expenditure which was not observed in normal weight sickle cell children. While it is possible that lowering resting energy expenditure with supplemental Gln may be an effective way to improve growth of children with sickle cell disease, the study design (i.e., lack of control group) underscores the need for large randomized controlled trials to test this hypothesis. Future studies should also focus on specific subgroups of sickle cell disease children, in whom Gln utilization may exceed endogenous Gln *de novo* synthesis or exogenous supply, resulting in Gln depletion, for example, when sickle cell disease children suffer from acute intercurrent illness or malnutrition.

#### 3.7.3. Enteral Glutamine Supplementation in Children with Cystic Fibrosis

Cystic fibrosis in children is often associated with malnutrition and poor growth. Chronic malnutrition in combination with frequent infections that are linked with acute stress may lead to depletion of body Gln stores in cystic fibrosis. Compared with normal children, neutrophil Gln deficiency has been observed in cystic fibrosis children [223]. Moreover, cystic fibrosis children with severe mutations showed even lower neutrophil Gln content compared to those with mild mutations [223]. Because Gln is a free amino acid, Darmaun et al. [224, 225] hypothesized that Gln should be readily absorbed in cystic fibrosis children with malabsorption secondary to exocrine pancreatic insufficiency and maybe of potential benefit.

In this one trial [225] (Table 7), Leu and Gln kinetics were measured in the postabsorptive state in 9 prepubertal (age 9.6 ± 0.5 y) children with cystic fibrosis who were either malnourished or short stature to test whether 4 weeks of oral Gln (0.7 g/kg/d), subcutaneous human recombinant growth hormone (rhGH; 0.3 mg/kg/week), or a combination of both agents (given in a random order) altered protein and Gln metabolism. Compared to baseline, 4-week oral Gln increased plasma Gln concentrations, whereas protein or Gln metabolism was not affected. In contrast, rhGH (as well as the combination of Gln+rhGH) reduced Leu oxidation and increased nonoxidative Leu disposal (an index of protein synthesis) with no change in Leu release from proteolysis. rhGH did not alter whole-body Gln metabolism (rate of Gln appearance, Gln release from proteolysis, or Gln *de novo* synthesis). Gln or rhGH given individually were associated with slight increases in lean body mass. Whereas Gln alone did not alter glucose metabolism, the increase in glucose and insulin/glucose ratio after rhGH treatment were blunted when rhGH was combined with oral Gln. Although oral Gln had no measurable protein anabolic effects in the fasting state in prepubertal cystic fibrosis children who are either malnourished or short, it is worth noting that Leu oxidation decreased in 6 out of 9 patients after Gln supplementation. Moreover, only 9 patients (of the12 recruited) completed the study. Hence, in addition to the limited statistical power, the wide intersubject variability of this heterogeneous population may have precluded the detection of Gln's effect.

Data from this one small study is not sufficient to determine whether Gln supplementation provides any benefit in cystic fibrosis children. Further study is needed. In addition to outcomes such as protein metabolism, glucose metabolism, and body composition, it may be worthwhile to test the effect of Gln on other outcomes, for example, immune function. Future studies should also focus on specific subgroups of this heterogeneous population, who may be Gln deficient, for example, children with severe cystic fibrosis mutations.

#### 3.7.4. Enteral Glutamine Supplementation in Children with Type 1 Diabetes

Although Gln is thought to be a major source of carbon for gluconeogenesis [66], results are conflicting on whether Gln impairs or accelerates recovery from hypoglycaemia [226–228]. A recent randomized crossover pilot study in children with type 1 diabetes has investigated whether oral Gln could improve hypoglycaemia during exercise and overnight after exercise [229]. Ten adolescents (mean age 15.2 ± 1.4 y) on insulin pumps were randomized to receive a drink containing Gln or placebo preexercise and at bedtime (0.25 g/kg/dose). Blood glucose was monitored hourly overnight. Fasting plasma Gln and ammonia concentrations were measured the following morning 16 h postexercise. Subjects returned within 3 weeks for an identical study with the reverse treatment. Blood glucose concentrations showed a similar % drop from baseline during exercise in Gln and placebo groups. Although a comparable number of subjects developed hypoglycaemia during exercise on Gln or placebo, postexercise overnight hypoglycaemia was more frequent after Gln than after placebo (≤70 mg/dL: Gln, 19%; placebo, 15%; *P* < 0.05; ≤60 mg/dL: Gln, 7.7%; placebo, 3.6%; *P* < 0.05). The cumulative probability of overnight hypoglycaemia was increased on the Gln day versus the placebo day (80% versus 50%, *P* < 0.05). Whereas plasma Gln concentrations were higher than the morning after Gln administration as compared with placebo (316 *μ*mol/L versus 200 *μ*mol/L, *P* < 0.001), plasma ammonia concentrations were similar. This pilot data suggests that Gln supplementation increases the likelihood of postexercise overnight hypoglycaemia in adolescents with type 1 diabetes. Results, however, are limited given the small sample size and lack of direct measurement of insulin sensitivity. Further study is needed in larger numbers of children with type 1 diabetes to determine whether Gln affects peripheral or hepatic insulin sensitivity.

## 4. Conclusions

### 4.1. Premature Neonates

Although parenteral Gln appears to have a protein-sparing effect in premature infants of LBW [80, 105], randomized trials and a meta-analysis of 7 high quality trials cannot confirm any consistent benefit(s) of enteral or parenteral Gln on clinical outcomes (e.g., mortality, length of stay, feeding tolerance, infections/sepsis, NEC, ventilator use, growth, or neurological sequelae) [99, 100, 102, 107, 111, 113, 129]. Further, the beneficial effects of parenteral Gln on protein metabolism were not reported with enteral Gln supplementation [125, 127], likely because the majority of enteral Gln is used in first pass in premature infants [126]. Although several high quality trials have studied the effects of enteral and parenteral Gln in premature neonates, results remain indeterminate. There are a number of explanations for this. Firstly, the choice of clinically relevant outcomes (e.g., rare events, like mortality) that are not influenced by other factors is challenging in a population of infants with rapidly changing clinical status. Furthermore, the differences in nutritional support regimens; different effects of enteral versus parenteral Gln supplementation could also contribute to the conflicting results. In addition to the route of administration (enteral versus parenteral), differences in Gln dose, supplement duration, studies varied with respect to initiation, advancement, and tapering of parenteral amino acids as well as the total daily intake. This in combination with the different practices for introducing or withholding EN and the variable breast milk intake limits comparisons between studies and could represent sources of confounding within a particular study. For example, advancement of amino acids (and hence Gln) may be less aggressive in sicker infants, the subgroup that may be more likely to benefit from exogenous Gln. Finally, the heterogeneous study population of premature neonates with different gestational age, birth weight, and varying degrees of acute illness may also affect outcome.

### 4.2. Older Infants and Children with Disease

In older infants and children with disease, fewer high-quality randomized trials have studied the effects of supplemental Gln in any one specific population. In infants with surgical gastrointestinal disease, data from 2 randomized trials and a meta-analysis are insufficient to determine whether enteral or parenteral Gln confers clinically significant benefits (feeding tolerance or intestinal permeability) [130–132]. Apart from this one trial [131], the effects of parenteral Gln supplementation have not been studied in older children with disease. In children suffering from diarrhea or malnutrition, results from 6 randomized trials are conflicting regarding the efficacy of oral Gln in the treatment of diarrheal disease or in improving intestinal barrier function or growth [160–162, 167–169]. In pediatric cancer patients undergoing chemotherapy, autologous BMT, and HSCT, results from 3 randomized trials suggest that oral Gln may reduce the severity and duration of oral mucositis [178–180]. However, preliminary studies in children with solid tumors are conflicting [181, 182]. Finally in DMD children, oral Gln inhibits whole-body proteolysis but does not appear to improve muscle strength or function (based on results from 3 small randomized trials) [205, 206, 209].

Studies in other childhood diseases are limited. In older children with gastrointestinal disease, firm conclusions cannot be made from 1 small randomized trial on the efficacy of enteral Gln on remission rates in pediatric patients with active Crohn's disease [148]. Furthermore, there is a lack of good quality data specific to pediatric patients with SBS to suggest any benefit of supplemental Gln in promoting intestinal adaptation, since studies are limited to case series [140–142]. Except for 1 small randomized trial in severely burned children showing no beneficial effects of enteral Gln on whole-body protein accretion [190], the 2 trials in pediatric and adult patients with severe burns/trauma had significant methodological limitations (high risk of bias) [195, 196]. And insufficient evidence is available to confer any beneficial effect(s) of Gln supplementation in chronic diseases of childhood. Other than one small randomized crossover in diabetic adolescents [229], data are limited to time series studies in cystic fibrosis and sickle cell anemia [222, 225].

Given the conflicting results (e.g., in premature infants) and insufficient data in other childhood conditions, the routine use of supplemental Gln cannot be recommended in any one pediatric population as a whole. It seems that the benefits of Gln supplementation are limited to specific subgroups of pediatric patients who may suffer from Gln deficiency, whereby Gln utilization exceeds the body's synthetic capacity or exogenous supply. Although mechanisms of Gln action have been proposed, there is still a need for fundamental research to better define the role of Gln in different life stages of childhood and to determine how Gln modulates cell-specific functions during health and disease processes. A better understanding of the mechanisms of Gln will help to eventually identify the subpopulations of pediatric patients for which Gln may (or may not) be beneficial. Given the abundant evidence demonstrating safety in all conditions studied so far, eventual evaluation in specific subgroups of children is warranted. However, the methodological issues noted from previous trials should be seriously considered in any future large randomized controlled trial involving Gln supplementation in sick children.

## Figures and Tables

**Table 1:** Glutamine-supplemented parenteral nutrition in premature neonates.

Reference	Subjects	Design	Gln	Control	Outcomes	Results
Lacey et al. 1996 [99]	44 VLBW premature neonates age <4 d receiving PN for <3 d; birth wt: 530–1250 g; GA <32 wk	Randomized double blind	Isonitrogenous isocaloric PN supplemented with Gln (15–25% of AA mix) for 14 ± 6 d (*n* = 22)	Standard PN for 16 ± 10 d (*n* = 22)	Time to full EN, d on PN, d on ventilator, LOS, other clinical outcomes and safety monitored throughout hospitalisation	No differences for entire cohort; birth wt <800 g subgroup had fewer d on PN, fewer d to full feeds, fewer d on ventilator, higher lymphocyte count, no difference in NICU LOS, safe
						
Des Robert et al. 2002 [105]	13 LBW age <3 d receiving exclusive PN; birth wt: 820–1650 g; GA: 28–30 wk	Randomized double blind	Gln-supplemented 0.5 g/kg/d exclusive PN started on d3 of life for 24 h, AA intake at 1.5 g/kg/d by d3 (*n* = 6)	Isonitrogenous Gln-free AA (Primène) supplemented exclusive PN started on d3 of life for 24 h, AA intake at 1.5 g/kg/d by d3 (*n* = 7)	Whole body protein metabolism/Leu kinetics (IV infusions of NaH13CO3 and L-[1–13C]Leu) on d4 of life during continuous PN (fed state)	Decreased rates of Leu release from protein breakdown and Leu oxidation, decreased rates of nonoxidative Leu disposal (an index of whole-body protein synthesis), safe
						
Thompson et al. 2003 [100]	35 ELBW ill premature neonates age <1 d; birth wt: <1000 g	Randomized double blind	Standard isonitrogenous isocaloric PN supplemented with 16% of AA as Gln started on d1 of life, AA intake started at 1.0 g/kg/d to ≤3.0 g/kg/d (*n* = 17)	Standard PN started on d1 of life, AA intake started at 1.0 g/kg/d to ≤3.0 g/kg/d (*n* = 18)	(1) Feeding tolerance; (2) growth, age at discharge, infection, number of episodes of culture-positive sepsis or NEC, metabolic tolerance and safety monitored until expected date of delivery, discharge or death (whichever came first)	(1) Fewer d to reach full EN; (2) no differences in gastric residuals, d to regain birth wt, wt gain, infection, total white cell count, episodes of low white cell count, number of episodes of culture-positive sepsis or NEC, age at discharge or death, well tolerated and safe
						
Poindexter et al. 2004* [102]	1433 ELBW (≤72 h after birth); birth wt: 401–1000 g; GA: 26 ± 2 wk	Multicentre-randomized double blind	Isonitrogenous study AA solution with 20% of AA as Gln in PN until 120 d of age, death, or discharge (whichever came first), AA intake ≤3–3.5 g/kg/d (*n* = 721)	Standard AA solution without Gln (TrophAmine) in PN until 120 d of age, death or discharge (whichever came first), AA intake ≤3–3.5 g/kg/d (*n* = 712)	(1) Death or late onset sepsis; (2) number of episodes of late onset sepsis, NEC, d on ventilator, LOS in hospital, tolerance of enteral feeds, feeding intolerance, growth, d of PN, safety	(1) No differences in death or late onset sepsis (culture after 72 h of age), (2) no differences in number of episodes of late onset sepsis, NEC, d on ventilator, LOS in hospital, d to first/full enteral feeds, incidence/number of episodes of feeding intolerance, d to reach 1500 g or wt at 36 wk postmenstrual age, increased d of PN, well tolerated and safe
						
Poindexter et al. 2003* [103]	141 ELBW (≤72 h after birth); birth wt: 401–1000 g; GA: 26 ± 2 wk	Multicentre-randomized double blind	Isonitrogenous study AA solution with 20% of AA as Gln in PN for ~10 d, AA intake ≤3–3.5 g/kg/d (*n* = 72)	Standard AA solution without Gln (TrophAmine) in PN for ~10 d, AA intake ≤3–3.5 g/kg/d (*n* = 69)	Safety as assessed by plasma concentrations of AA and ammonia after infants had received study PN (2.3 ± 1.0 g/kg/d AA) for ~10 d	Increased plasma Gln (Gln group only) with no apparent biochemical risk, increased plasma essential AA (both groups) whereas Phe and Tyr decreased with greater decrease in Tyr (Gln group), no change in plasma ammonia
						
Kalhan et al. 2005 [80]	20 LBW clinically stable (24–48 h after birth); birth wt: 694–1590 g; GA ≤32 wk	Randomized double blind	Isonitrogenous AA mixture supplemented with Gln 0.6 g/kg/d in PN for 3–5 d, AA intake ~3.0 g/kg/d (*n* = 10)	AA mixture without Gln in PN for 3–5 d, AA intake ~3.0 g/kg/d (*n* = 10)	Whole body protein and Gln kinetics (IV infusions of [2H5]Phe, L-[1–13C,15N]Leu, [15N2]urea, L-[5–15N]Gln) on d6 or d7 of life while receiving AA mixture continuously after 3–5 d	Lower endogenous rates of appearance of Phe and Leu N (indices of proteolysis), lower Gln de novo synthesis, no differences in rate of appearance of Leu C, urea turnover or plasma AA concentrations
						
Li et al. 2007 [101]	53 LBW premature infants (48–72 h after birth); birth wt: <2000 g; GA <37 wk	Prospective intervention	Isonitrogenous AA solution with 20% of AA content as Alanyl-Gln dipeptide in PN until >2 wk, AA intake started at 0.5–1.0 g/kg/d to ≤3.0 g/kg/d (*n* = 28)	Routine PN until >2 wk, AA intake started at 0.5–1.0 g/kg/d to ≤3.0 g/kg/d (*n* = 25)	Growth, biochemical indices, feeding tolerance, and infective episodes throughout hospitalization	Fewer d to regain birth wt, no differences in wt or head circumferences, fewer d on PN, fewer episodes of hospital-acquired infection, shorter LOS in hospital, safe
						
Wang et al. 2010 [104]	30 VLBW; median age (interquartile range) (d): 2.5(1.0-2.0) (Gln) and 2.2(1.0-2.0) (control); birth wt: <1500 g; PN for ≥7 d; mean GA (wk) ± SD: 31.3 ± 1.5 (Gln) and 30.5 ± 1.8 (control)	Multicentre-randomized double blind	6% pediatric AA compound injection, average AA dosage 1.7 g/kg/d with Gln 0.3 g/kg/d, PN decreased when EN increased and PN withheld when >70% of recommended intake from EN (*n* = 13)	6% pediatric AA compound injection, average AA dosage 2.0 g/kg/d, PN decreased when EN increased and PN withheld when >70% of recommended intake from EN (*n* = 15)	(1) Mortality and changes in hepatic function (bile acid, ALT, AST, total bilirubin, direct bilirubin, prealbumin, albumin), (2) time to full EN (d), episodes of gastric residual, total duration of PN (d), wt gain (g/d), head circumference (cm), LOS, d on ventilator	(1) Decreased AST and direct bilirubin, no differences in bile acid, ALT, total bilirubin, prealbumin, or albumin, (2) no differences in time to full EN, episodes of gastric residual, total duration of PN, wt gain, head circumference, LOS or d on ventilator

VLBW: very low birth weight; PN: parenteral nutrition; wt: weight; GA: gestational age; AA: amino acid; EN: enteral nutrition; LOS: length of stay; NICU: neonatal intensive care unit; LBW: low birth weight; IV: intravenous; ELBW: extremely low birth weight; NEC: necrotizing enterocolitis; AST: aspartate aminotransferase; ALT: alanine aminotransferase.

*Originating from the same cohort.

**Table 2:** Glutamine-supplemented enteral nutrition in premature neonates.

Reference	Subjects	Design	Gln	Control	Outcomes	Results
Neu et al. 1997* [107]	68 VLBW age <3 d receiving PN; birth wt: 500–1250 g; GA: 24–32 wk	Randomized double-blind	From d3 to d30 of life received premature formula (Similac Special Care Group) supplemented with Gln at 0.08 g/kg/d on d3 and reached 0.31 g/kg/d by d13, PN AA started at 0.5 g/kg/d on d3 until 3 g/kg/d by d8 (*n* = 35)	From d3 to d30 of life received premature formula (Similac Special Care Group) alone, PN AA started at 0.5 g/kg/d on d3 until 3 g/kg/d by d8 (*n* = 33)	(1) Hospital-acquired sepsis, tolerance to enteral feedings, and LOS in hospital; (2) analysis of T-cell subsets in peripheral blood during wk-4 of life, NEC, growth, respiratory status, and safety	(1) Reduced hospital-acquired sepsis (positive blood culture) when controlled for birth wt, fewer % d with no oral intake, and no differences in LOS in hospital; (2) blunted the rise in HLA-DR+ and CD16/CD56 subsets, no differences in NEC, wt, length, head circumference, mechanical ventilation or death, safe
Roig et al. 1996* [108]	68 VLBW age <3 d receiving PN; birth wt: 500–1250 g; GA: 25–32 wk	Randomized double-blind	From d3 to d30 of life received premature formula (Similac Special Care Group) supplemented with Gln at 0.08 g/kg/d on d3 and reached 0.31 g/kg/d by d13, PN AA started at 0.5 g/kg/d on d3 until 3 g/kg/d by d8 (*n* = 35)	From d3 to d30 of life received premature formula (Similac Special Care Group) alone, PN AA started at 0.5 g/kg/d on d3 until 3 g/kg/d by d8 (*n* = 33)	Plasma AA concentrations at 3 time points (prefeeding, transition to full feeds and full EN)	Decreased plasma concentrations of Ala, Gly, Ser, Thr, Phe, and total nonessential AA after ~2 wk supplementation (i.e., at transition to full EN), no differences between groups for plasma concentrations of Gln, Glu, or ammonia during study
Darmaun et al. 1997* [125]	11 VLBW age <3 d receiving PN; birth wt: <1250 g; GA: 24–32 wk	Randomized double-blind	Received premature formula (Similac Special Care Group) supplemented with Gln at 0.08 g/kg/d on d3 and increased to 0.2 g/kg/d on d10, PN AA started at 0.5 g/kg/d on d3 until 3 g/kg/d by d8 (*n* = 6)	Received premature formula (Similac Special Care Group) alone on d3 of life until d10, PN AA started at 0.5 g/kg/d on d3 until 3 g/kg/d by d8 (*n* = 5)	Whole body protein metabolism/Leu kinetics and Gln kinetics (IV infusions of L-[2H]Leu and L-[13C]Gln) on d10 of life in fed state, that is, during continuous EN (Gln supplemented or nonsupplemented) and PN	No differences in overall rate of appearance of Leu and Gln, no differences in Leu and Gln release from protein breakdown, no differences in Gln *de novo* synthesis or plasma Gln concentrations
Dallas et al. 1998* [109]	68 VLBW age <3 d receiving PN; birth wt: 500–1250 g; GA: 24–32 wk	Randomized double-blind	From d3 to d30 of life received premature formula (Similac Special Care Group) supplemented with Gln at 0.08 g/kg/d on d3 and reached 0.31 g/kg/d by d13, PN AA started at 0.5 g/kg/d on d3 until 3 g/kg/d by d8 (*n* = 35)	From d3 to d30 of life received premature formula (Similac Special Care Group) alone, PN AA started at 0.5 g/kg/d on d3 until 3 g/kg/d by d8 (*n* = 33)	Hospital costs analysed by log-rank tests and Kaplan-Meier plots	Reduced median costs for hospitalisation, radiology, pharmacy, laboratory, and NICU, reduced median number of utilization units
Barbosa et al. 1999 [110]	9 critically ill infants aged 1–24 mo requiring intensive care and tolerating EN	Randomized double-blind	Gln supplemented 0.3 g/kg/d EN for 5 d (*n* = 5)	Casein supplemented 0.3 g/kg/d EN for 5 d (*n* = 4)	Septic morbidity, mortality, LOS in ICU, LOS in hospital and duration of mechanical ventilation, and tolerance	No significant differences in septic complications, mortality, d in ICU, d in hospital or d with ventilation, well tolerated, and safe
Mercier et al. 2003 [112]	25 LBW premature neonates age ≥14 d without acute illness and receiving exclusive EN; birth wt: 980–1890 g; GA: 27–35 wk	Randomized double-blind	Exclusive enteral preterm infant formula supplemented with Gln 0.7 g/kg/d (17% of total protein intake as Gln) for 21 d (*n* = 12)	Exclusive enteral preterm infant formula supplemented with isonitrogenous lactoserum extracted protein 0.7 g/kg/d (17% of total protein intake as lactoserum) for 21 d (*n* = 9)	Parameters of superior mesenteric blood flow velocities measured in fed state by pulsed Doppler ultrasound after 21 d supplementation	No effect on superior mesenteric blood flow
Vaughn et al. 2003 [111]	649 VLBW age <7 d receiving PN; birth wt: 500–1250 g	Multicentre randomized double-blind	Enteral Gln supplement (0.3 g/kg/d, 3% Gln in sterile water) given at the same time but separate from feedings for the first 28 d of life; AA intake ~3–3.5 g/kg/d by d7 (*n* = 314)	Enteral placebo (sterile water) given at the same time but separate from feedings for the first 28 d of life; AA intake ~3–3.5 g/kg/d by d7 (*n* = 335)	(1) Number of episodes of blood culture-proven nosocomial sepsis between 7 d to 36 wk of age; (2) suspected sepsis, pneumonia, UTI, meningitis, NEC, IVH, PVL, retinopathy of prematurity, use of oxygen at 36 wk, gastrointestinal dysfunction, growth, age and weight at discharge, and death	(1) No differences in nosocomial sepsis; (2) decreased gastrointestinal dysfunction and severe neurologic sequelae among survivors (Grades 3 and 4 IVH and PVL), no differences in suspected sepsis, pneumonia, UTI, meningitis, NEC, retinopathy of prematurity, use of oxygen at 36 wk, growth, age and wt at discharge, or death
Parimi et al. 2004 [127]	26 LBW in good health and gaining wt aged 10–74 d (>23 d of age); birth wt: 693–1846 g; GA <32 wk	Randomized double-blind	Enteral Gln 0.6 g/kg/d for 5 d (*n* = 9)	(1) Enteral Gly 0.6 g/kg/d for 5 d (*n* = 9) or (2) unsupplemented enteral formula for 5 d (*n* = 8)	Whole-body protein, Gln and urea kinetics on study d-6 (IV infusions of L-[1–13C,15N]Leu, [2H5]Phe, [15N2]urea, L-[5–15N]Gln) after 5 d supplementation during fasting (3 h after the last meal) and fed state, and plasma AA concentrations during fasting	Higher rate of urea synthesis, no differences in rates of appearance of Phe, Leu C or Leu N, no differences in rate of appearance of Gln or plasma Gln concentrations
Korkmaz et al. 2007 [123]	69 VLBW premature neonates receiving PN; birth wt: <1500 g	Prospective intervention	From 8 d to 120 d of life received Gln supplement 0.15 g/kg/d BID mixed with sterile water and given at the same time but separate from feedings by an orogastric tube or orally (*n* = 36)	From 8 d to 120 d of life received sterile water (placebo) given at the same time but separate from feedings by an orogastric tube or orally (*n* = 33)	Growth parameters at birth, 1, 2, 3, and 4 mo and biochemical parameters at 4 mo	No differences in growth parameters at birth and 2 mo, higher wt, length, head circumference, MUAC and mid-thigh circumference at 3 and 4 mo, no toxicity signs
Van Den Berg et al. 2005** [113]	102 VLBW <48 h after birth receiving PN; birth wt: <1500 g; GA <32 wk	Randomized double-blind	From 3 d to 30 d of life received enteral preterm formula or breast milk supplemented with Gln in increasing doses to ≤0.3 g/kg/d (*n* = 52)	From 3 d to 30 d of life received enteral preterm formula or breast milk supplemented with isonitrogenous Ala in increasing doses to ≤0.3 g/kg/d (*n* = 50)	(1) Feeding tolerance; (2) growth, infectious morbidity, and short-term outcome	(1) No differences in time to full enteral feeding; (2) no differences in age PN discontinued, d of no enteral feeding, NEC or wt z-scores, decreased incidence of ≥1 serious infections (sepsis, meningitis, pyelonephritis, pneumonia, and arthritis), no differences in PDA treated with indomethacin or surgical ligation, mechanical ventilation, supplemental oxygen, retinopathy, age at discharge from NICU, age at discharge from hospital or death
Van Den Berg et al. 2005** [114]	102 VLBW <48 h after birth receiving PN; birth wt: <1500 g; GA <32 wk	Randomized double-blind	From 3 d to 30 d of life received enteral preterm formula or breast milk supplemented with Gln in increasing doses to ≤0.3 g/kg/d (*n* = 52)	From 3 d to 30 d of life received enteral preterm formula or breast milk supplemented with isonitrogenous Ala in increasing doses to ≤0.3 g/kg/d (*n* = 50)	Safety by determination of plasma AA concentrations measured at 4 time points: <48 h, d7, d14, and d30 of life	Increased plasma AA concentrations of most essential and nonessential AA including Gln and Glu (both groups), decreased Phe and Tyr (both groups)
Van Den Berg et al. 2006** [116]	90 VLBW <48 h after birth receiving PN; birth wt: <1500 g; GA <32 wk	Randomized double-blind	From 3 d to 30 d of life received enteral preterm formula or breast milk supplemented with Gln in increasing doses to ≤0.3 g/kg/d (*n* = 45)	From 3 d to 30 d of life received enteral preterm formula or breast milk supplemented with isonitrogenous Ala in increasing doses to ≤0.3 g/kg/d (*n* = 45)	Intestinal permeability (urinary lactulose/mannitol ratio) assessed at 4 time points: <48 h, d7, d14, and d30 of life	Decreased lactulose/mannitol ratio (both groups), decreased urinary lactulose concentrations (both groups), increased urinary mannitol concentrations (both groups)
Van Den Berg et al. 2007** [115]	86 VLBW <48 h after birth receiving PN; birth wt: <1500 g; GA <32 wk	Randomized double-blind	From 3 d to 30 d of life received enteral preterm formula or breast milk supplemented with Gln in increasing doses to ≤0.3 g/kg/d (*n* = 43)	From 3 d to 30 d of life received enteral preterm formula or breast milk supplemented with isonitrogenous Ala in increasing doses to ≤0.3 g/kg/d (*n* = 43)	Intestinal (faecal) microflora (bifidobacteria, lactobacilli, Escheria coli, streptococci, clostridia) at <48 h, at d7, d14, and d30 of life, as analysed by fluorescent in situ hybridization	No difference in prevalence of intestinal microflora
Van Den Berg et al. 2007** [117]	77 VLBW surviving infants at the corrected age of 1 y; birth wt: <1500 g; GA <32 wk	Followup of all surviving participants of a randomized double-blind study	From 3 d to 30 d of life received enteral preterm formula or breast milk supplemented with Gln in increasing doses to ≤0.3 g/kg/d (*n* = 37)	From 3 d to 30 d of life received enteral preterm formula or breast milk supplemented with isonitrogenous Ala in increasing doses to ≤0.3 g/kg/d (*n* = 40)	Incidence of allergic and infectious disease during the first year of life as assessed by validated questionnaires	Lower risk of atopic dermatitis, no differences in incidence of bronchial hyperactivity, infections of upper respiratory, lower respiratory, or gastrointestinal tracts
Van Zwol et al. 2008** [122]	72 VLBW infants at the corrected age of 2 y; birth wt: <1500 g; GA <32 wk	Followup of participants of a randomized double-blind study	From 3 d to 30 d of life received enteral preterm formula or breast milk supplemented with Gln in increasing doses to ≤0.3 g/kg/d (*n* = 40)	From 3 d to 30 d of life received enteral preterm formula or breast milk supplemented with isonitrogenous Ala in increasing doses to ≤0.3 g/kg/d (*n* = 32)	Neurodevelopmental outcome (neurodevelopmental impairment, cerebral palsy, vision, hearing, MDI, and PDI of the Bayley Scale of Infant Development II) evaluated at the corrected age of 2 y	No differences in the incidence of an MDI nor a PDI ≤ 85, no differences in the incidence of neurodevelopmental impairment or cerebral palsy
Van Den Berg et al. 2009** [120]	63 VLBW infants <48 h after birth receiving PN; birth wt: <1500 g; GA <32 wk	Randomized double-blind	From 3 d to 30 d of life received enteral preterm formula or breast milk supplemented with Gln in increasing doses to ≤0.3 g/kg/d (*n* = 25)	From 3 d to 30 d of life received enteral preterm formula or breast milk supplemented with isonitrogenous Ala in increasing doses to ≤0.3 g/kg/d (*n* = 38)	Th1 (IF-gamma, TNF-alpha, IL-2) and Th2 cytokine responses (IL-10, IL-5, IL-4) at 48 h, d7, and d14 of life following in vitro whole blood cell stimulation as analyzed by cytometric bead array	No differences in Th1 or Th2 cytokine responses
Van Zwol et al. 2009** [119]	59 VLBW infants at age of 1 y; birth wt: <1500 g; GA <32 wk	Followup of participants of a randomized double-blind study	From 3 d to 30 d of life received enteral preterm formula or breast milk supplemented with Gln in increasing doses to ≤0.3 g/kg/d (*n* = 28)	From 3 d to 30 d of life received enteral preterm formula or breast milk supplemented with isonitrogenous Ala in increasing doses to ≤0.3 g/kg/d (*n* = 31)	Th1 (IF-gamma, TNF-alpha, IL-2) and Th2 cytokine profiles (IL-10, IL-5, IL-4) at 1 y of age following in vitro whole blood stimulation	No differences in Th1 or Th2 cytokine profiles
Van Zwol et al. 2010** [121]	72 VLBW infants at the corrected age of 1 y; birth wt: <1500 g; GA <32 wk	Followup of participants of a randomized double-blind study	From 3 d to 30 d of life received enteral preterm formula or breast milk supplemented with Gln in increasing doses to ≤0.3 g/kg/d (*n* = 34)	From 3 d to 30 d of life received enteral preterm formula or breast milk supplemented with isonitrogenous Ala in increasing doses to ≤0.3 g/kg/d (*n* = 38)	Intestinal (faecal) microbiota (bifidobacteria, Chis/lit group, Escheria coli) at age 1 y as analysed by fluorescent *in situ* hybridization	No difference in prevalence of intestinal microbiota
Van Zwol et al. 2011** [118]	76 VLBW infants at 6 y of age; birth wt: <1500 g; GA <32 wk	Followup of participants of a randomized double-blind study	From 3 d to 30 d of life received enteral preterm formula or breast milk supplemented with Gln in increasing doses to ≤0.3 g/kg/d (*n* = 38)	From 3 d to 30 d of life received enteral preterm formula or breast milk supplemented with isonitrogenous Ala in increasing doses to ≤0.3 g/kg/d (*n* = 38)	Allergic (atopic dermatitis, hay fever, recurrent wheeze, and asthma) and infectious disease (upper and lower respiratory tract infection, gastrointestinal tract infection, urinary tract infection) at 6 y of age as assessed by validated questionnaires	Decreased risk of atopic dermatitis, no differences in hay fever, recurrent wheeze or asthma, decreased risk of gastrointestinal tract infection, no differences in upper or lower respiratory tract infection or urinary tract infection

VLBW: very low birth weight; PN: parenteral nutrition; wt: weight; GA: gestational age; AA: amino acid; EN: enteral nutrition; IV: intravenous; LOS: length of stay; NEC: necrotizing enterocolitis; ICU: intensive care unit; LBW: low birth weight; UTI: urinary tract infection; IVH: intraventricular hemorrhage; PVL: periventricular leukomalacia; PDA: patent ductus arteriosus; NICU: neonatal intensive care unit; BID: twice a day; MUAC: mid upper arm circumference; MDI: Mental Developmental Index; PDI: Psychomotor Developmental Index; Th1: T helper type 1; Th2: T helper type 2; IF: interferon; TNF: tumor necrosis factor; IL: interleukin.

*, **Originating from the same cohort.

**Table 3:** Glutamine supplementation in pediatric patients with gastrointestinal disease.

Reference	Subjects	Design	Gln	Control	Outcomes	Results
Albers et al. 2005 [131]	80 newborns and infants age ≤2 y requiring PN after major digestive tract surgery (GA ≥30 wk)	Randomized double-blind	Parenteral Gln; isonitrogenous isocaloric Gln (≤0.4 g/kg/d) supplemented PN started on d-2 after surgery and reached 90% of recommended AA intake of 1.5–2.5 g/kg/d (90% of Gln target dose of 0.4 g/kg/d) by d-4 until d-31, full EN, discharge or death; with tapering of PN started on or after d-6 (*n* = 41)	Standard PN started on d-2 after surgery and reached 90% of recommended AA intake of 1.5–2.5 g/kg/d by d-4 until d-31, full EN discharge or death, with tapering of PN started on or after d-6 (*n* = 39)	(1) Intestinal permeability (urinary lactulose/rhamnose ratio measured wk-1 through -4 after surgery, (2) nitrogen balance on d-4, -5, -6, urinary 3-MH excretion on d-5, mortality, LOS in ICU and in hospital, septic episodes, usage of antibiotics and ICU resources	(1) No effect on intestinal permeability, (2) no effect on nitrogen balance, urinary 3-MH excretion, mortality, LOS in ICU or in hospital, culture-proven sepsis, usage of antibiotics or ICU resources, and no adverse effects
Duggan et al. 2004 [130]	20 neonates and infants aged <12 mo requiring PN after surgery for congenital or acquired gastrointestinal disease	Randomized double-blind	Enteral Gln; breastmilk or protein hydrolysate formula supplemented with enteral Gln (≤0.4 g/kg/d) until 7 consecutive d of full EN or hospital discharge (*N* = 9)	Breastmilk or protein hydrolysate formula supplemented with enteral iso-osmolar mix of nonessential AA (≤0.4 g/kg/d) until 7 consecutive d of full EN or hospital discharge (*N* = 11)	(1) Duration (d) on PN and d to achieve enteral feedings providing ≥80% of US-recommended dietary allowance for energy, (2) changes in macronutrient and energy absorption after supplementation, growth, and frequency of infections during trial	(1) No differences in d on PN or d to achieve ≥80% energy requirements by EN, (2) no improvement in energy absorption, no differences in frequency of infections, wt gain, length gain, or changes in anthropometric measures, safe
Zhu et al. 2002 [142]	27 patients aged 9–67 y with SBS	Case series	Enteral/parenteral Gln; enteral and/or parenteral glycyl-Gln powder (0.6 g/kg/d) for ~1.5 ± 1.0 y or alanyl-Gln solution (0.3 g/kg/d) in combination with 3 wk rhGH and rehabilitative diet	None	Nutritional status and intestinal absorptive capacity after an average of 1.5 ± 1.0 y treatment	Increased wt, serum total protein, albumin, hemoglobin, reduced stool frequency and stool nitrogen, increased D-xylose absorption
Weiming et al. 2004 [141]	37 patients aged 9–74 y with SBS (including 6 children aged 9–13 y)	Case series	Enteral Gln; oral Gln powder (0.6 g/kg/d) for 1-2 y in combination with rhGH (0.05 mg/kg/d) for 3 wk and rehabilitative diet	None	Efficacy in weaning off PN, intestinal absorptive capacity (D-xylose absorption, stool frequency, stool nitrogen) and plasma protein concentrations after 2-3 y of treatment	Improved intestinal absorptive capacity and increased plasma protein concentrations, 57% of patients weaned off PN
Ladd et al. 2005 [140]	2 female pediatric patients aged 6 y (GA: 33 wk) and 6.5 y (GA: 26 wk) with SBS from neonatal gastrointestinal catastrophes	Retrospective review of medical records	Enteral Gln, SC rhGH (0.3 mg/kg/wk) and concurrent enteral Gln supplementation (30 mg/d) started at 6 y and 6.5 y, for 8 and 2.5 y, respectively	None	Retrospective review of supplemental nutritional requirement and serial growth parameters of height and wt over treatment period	Improvement in growth (approximating target percentiles for height and wt), improved intestinal tolerance/adaptation, weaned from PN
Akobeng et al. 2000* [148]	18 children aged 6.8–15.7 y with active Crohn's disease	Randomized double-blind	Enteral Gln, exclusive polymeric EN with 42% of AA content as Gln for 4 wk (*n* = 9)	Isocaloric isonitrogenous exclusive standard polymeric EN with identical essential AA and 4% of AA content as Gln for 4 wk (*n* = 9)	Remission rates after 4 wk exclusive EN, changes in clinical and laboratory parameters of disease activity during 4 wk exclusive EN	No differences in remission rates, changes in platelet count, orosomucoid level or wt, less improvement in pediatric Crohn's disease activity index
Akobeng et al. 2000* [150]	16 children aged 6.8–15.7 y with active Crohn's disease	Randomized double-blind	Enteral Gln, exclusive polymeric EN with 42% of AA content as Gln for 4 wk (*n* = 7)	Isocaloric isonitrogenous exclusive standard polymeric EN with identical essential AA and 4% of AA content as Gln for 4 wk (*n* = 9)	Intestinal permeability as assessed by lactulose/mannitol urinary excretion ratio after 4 wk exclusive EN	Decreased lactulose/mannitol intestinal permeability ratio (both groups)
Akobeng et al. 2002* [151]	15 children aged 6.8–15.7 y with active Crohn's disease	Randomized double-blind	Enteral Gln, exclusive polymeric EN with 42% of AA content as Gln for 4 wk (*n* = 7)	Isocaloric isonitrogenous exclusive standard polymeric EN with identical essential AA and 4% of AA content as Gln for 4 wk (*n* = 8)	Serum IGF-1 concentrations and growth after 4 wk exclusive EN	No improvement in serum IGF1 (both groups), less improvement in wt, wt for height and MUAC
Akobeng et al. 2007* [152]	15 children aged 6.8–15.7 y with active Crohn's disease	Randomized double-blind	Enteral Gln, polymeric EN with 42% of AA content as Gln for 4 wk (*n* = 7)	Isocaloric isonitrogenous exclusive standard polymeric EN with identical essential AA and 4% of AA content as Gln for 4 wk (*n* = 8)	Plasma antioxidant concentrations after 4 wk exclusive EN	Increased plasma selenium concentrations (both groups), decreased concentrations of vitamin C and E (both groups), no change in vitamin A, urates, glutathione, and malondialdehyde

PN: parenteral nutrition; EN: enteral nutrition; wt: weight; GA: gestational age; AA: amino acid; 3-MH: 3-methyl-histidine; LOS: length of stay; ICU: intensive care unit; SBS: short bowel syndrome; rhGH: recombinant growth hormone; SC: subcutaneous; IGF-1: insulin-like growth factor-1; MUAC: mid upper arm circumference.

*Originating from the same cohort.

**Table 4:** Enteral glutamine supplementation in children with diarrheal disease/malnutrition.

Reference	Subjects	Design	Gln	Control	Outcomes	Results
Ribeiro Jr. et al. 1994 [160]	118 male infants aged 1–12 mo with acute noncholera diarrhea and dehydration	Randomized double-blind	Oral Gln (90 mmol/L) added to standard glucose-based (90 mmol/L) WHO-ORS (*n* = 59)	Standard glucose-based (111 mmol/L) WHO-ORS (*n* = 59)	(1) Duration and severity (stool output, stool output rate) of diarrhea and (2) intake of ORS (recorded at 6 h intervals in a metabolic unit), urine output, vomitus, body wt, blood gases, and electrolytes monitored during hospitalization	(1) No differences in diarrheal stool output, stool output rate, or duration of diarrhea, (2) no differences in volume of ORS to achieve and maintain hydration, amount and number of vomitus, urine output, wt gain, safe, and well tolerated
						
Yalçin et al. 2004 [161]	128 otherwise healthy children aged 6–24 mo with acute (<10 d) diarrhea	Randomized double-blind	Oral Gln (0.1 g/kg/d TID) capsules dissolved in water for 7 d (*n* = 63)	Oral placebo (cornstarch) capsules dissolved in water for 7 d (*n* = 65)	(1) Duration of diarrhea, (2) severity of diarrhea by mother self-report followed until end of diarrheal episode, immunologic parameters 7 d after treatment, wt gain and infectious disease monitored monthly until 3 months after treatment	(1) Shorter duration of diarrhea for entire cohort and for age ≥12 mo subgroup, (2) no differences in frequency of persistent diarrhea or vomiting, no differences in serum IL-8, salivary immunoglobulin A, wt gain or frequency of infections
						
Gutierrez et al. 2007 [162]	147 dehydrated children aged 1–60 mo with acute noncholera diarrhea	Randomized double-blind	WHO-ORS in which glucose was replaced with Gln (20 g/L) until rehydration (*n* = 73)	Standard glucose-based WHO-ORS until rehydration (*n* = 74)	Efficacy in reducing (1) stool volume and (2) rehydratation time in acute diarrhea	(1) No differences in stool output during first 4 h, (2) no differences in time to successful rehydration, volume of ORS required for rehydration, urine output, or vomiting
						
Lima et al. 2005 [168]	80 malnourished (WAZ < −2) hospitalized children aged 2–60 mo with or without diarrhea	Randomized double-blind	Gln-supplemented (16.2 g/d) enteral modified WHO formula for 10 d (*n* = 26)	(1) Modified WHO enteral formula standard (*n* = 27 not randomized) or (2) isomolar Gly-supplemented (8.3 g/d) enteral modified WHO formula for 10 d (*n* = 27)	(1) Intestinal permeability (urinary lactulose/mannitol ratio), (2) wt gain after 10 d supplementation	(1) Improvement in intestinal barrier function (decreased lactulose/mannitol ratio versus Gly or standard), (2) no effect on % urinary excretion of lactulose or mannitol or on duration of diarrhea, no differences in wt gain, no improvement in WAZ, safe, and well tolerated
						
Lima et al. 2007 [169]	107 malnourished (WAZ, HAZ, or WHZ < −1) children aged 7.9–82.2 mo in northeastern Brazil	Randomized double-blind phase III	Oral alanyl-Gln (24 g/d) mixed with whole milk for 10 d (*n* = 51)	Oral isonitrogenous Gly (25 g/d) mixed with whole milk for 10 d (*n* = 56)	(1) Intestinal permeability (urinary lactulose/mannitol ratio) 10 d after supplementation, (2) growth (wt and height) measured until d120 of study, diarrheal disease morbidity	(1) No effect on lactulose/mannitol ratio, (2) improvement in barrier function (decrease in % lactulose recovery), reduction in absorptive epithelium (decrease in % mannitol recovery), improved (increased) cummulative change over 120 d in WHZ and WAZ but not HAZ (after adjustment for age and season), safe and well tolerated
						
Williams et al. 2007 [167]	93 malnourished growth-faltering Gambian infants aged 4–11 mo	Randomized double-blind	Oral Gln 0.25 g/kg/d BID mixed with expressed breastmilk or distilled water for 5-6 mo (rainy season) (*n* = 46)	Oral isonitrogenous isocaloric mix of nonessential AA (Ala, Gly, Ser, Asn; 0.25 g/kg/d BID) and fructose mixed with expressed breastmilk or distilled water for 5-6 mo (rainy season) (*n* = 47)	(1) Growth and intestinal barrier function (intestinal permeability) measured monthly during 5 mo supplementation and 6 mo after, (2) plasma markers of immunostimulation (immunoglobulins and acute phase proteins) during supplementation and morbidity (as reported by mother)	(1) No improvement in wt gain or length gain, no improvement in lactulose/mannitol intestinal permeability ratio or % urinary lactulose or mannitol recovery, (2) no effect on plasma concentrations of immunoglobulins or acute phase proteins or on morbidity

WHO: World Health Organization; ORS: oral rehydration solution; wt: weight; TID: 3 times a day; IL: interleukin; WAZ: weight for age z-score; HAZ: height for age z-score; WHZ: weight for height z-score; BID: twice a day; AA: amino acid.

**Table 5:** Glutamine supplementation in pediatric oncology patients.

Reference	Subjects	Design	Gln	Control	Outcomes	Results
Anderson et al. 1998 [180]	193 patients aged 1–62 y (72 children aged 1–18 y) undergoing BMT	Randomized double-blind	Enteral Gln; oral Gln suspension 1 g AA/m2 QID administered from admission until 28 d after BMT (*n* = 98; 34 children)	Oral Gly suspension 1 g AA/m2 QID administered from admission until 28 d after BMT (*n* = 95; 38 children)	(1) Severity and duration of stomatitis by self-report and by opiate use at d7, d14, and d28 after BMT, (2) PN use, rate of relapse or progression of malignancy, parental antibiotic use, acute or chronic GVHD, LOS in hospital, bacterial, or fungal infections, survival, and Gln toxicity during d0–d28 after BMT in patients prospectively stratified by type of transplant (autologous, matched sibling donor, or unrelated donor)	For autologous BMT: (1) reduced severity of oral mucositis by self report and reduced opiate use; for matched sibling BMT: no effect on severity of oral mucositis by self report, increased opiate use and (2) increased % survival at d28; for autologous or allogeneic BMT: no differences in PN use, rate of relapse or progression of malignancy, antibiotic use, acute or chronic GVHD, LOS in hospital, infections or survival, safe
Anderson et al. 1998 [179]	13 cancer patients (10 children aged 4–17 y) receiving chemotherapy	Randomized double-blind crossover	Enteral Gln; oral Gln suspension 2 g AA/m2 BID during chemotherapy and for ≥14 d after chemotherapy (*n* = 13; 10 children)	Oral Gly suspension 2 g AA/m2 BID during chemotherapy and for ≥14 d after chemotherapy (*n* = 13; 10 children)	Patient self-report of onset, duration, and severity of stomatitis over each chemotherapy course, as assessed by a questionnaire/calendar completed by the patient	Decreased number of d of mucositis, reduced severity (decreased number of d of oral mucositis ≥ Grade 2; Modified Eastern Cooperative Oncology Group, requiring restricted oral intake to soft foods), well tolerated
Pietsch et al. 1999 [175]	17 children aged 2–19 y receiving intensive chemotherapy (*n* = 14) or BMT (*n* = 3)	Case series	Enteral Gln; continuous nasogastric feedings of Gln-supplemented elemental EN (Vivonex Pediatric) during chemotherapy and at rehospitalization for ~12.7 d	None	Feasibility (hospital costs and complications)	Well-tolerated, lower hospital charges for enteral feedings compared to the same number of d of PN
Ward et al. 2003 [176]	13 pediatric patients aged 3–18 y undergoing treatment for malignancy	Phase 1 pharmaco-kinetic (initial dose-finding study)	Enteral Gln; oral Gln (mixed with sterile water) administered as 1 dose starting at 0.35 g/kg and increased by increments of 0.15 g/kg if 3 patients at each dose level tolerated the Gln	None	Patient acceptability and safety as assessed by measurement of plasma concentrations of Gln and ammonia at 60, 90, 120, 240, and 360 min after a single dose of Gln	Well tolerated and safe at 0.35, 0.5, and 0.65 g/kg; at 0.65 g/kg: plasma Gln and ammonia peaked between 30 and 90 min and returned to normal by 360 min post-Gln
Aquino et al. 2005 [178]	120 children [mean age (y) ± SEM: 8.9 ± 1.0 (Gln) and 10.5 ± 0.6 (Gly)] undergoing HSCT	Randomized double-blind	Enteral Gln; oral Gln 2 g/m2/dose BID (maximum dose 4 g) until 28 d posttransplant or discharge (*n* = 57)	Oral Gly 2 g/m2 BID (maximum dose 4 g) until 28 d posttransplant or discharge (*n* = 63)	(1) Oral mucositis graded by modified Walsh Scale, (2) maximum mucositis score, d of IV narcotic use, d of PN use, ≥1 episodes of bacteremia, LOS in hospital, mortality, and toxicity until discharge or until 28 d posttransplant	(1) Trend toward a reduction in mean mucositis score, (2) no difference in maximum mucositis score, reduced number of d of IV narcotic use, reduced d of PN, no differences in number of episodes of bacteremia, LOS in hospital or mortality, safe, and well tolerated
Okur et al. 2006 [181]	21 children aged 1–17 y with solid tumors receiving chemotherapy	Time series	Enteral Gln; oral Gln 2 g/m2/d BID during 5 d chemotherapy course (*n* = 21)	The same protocol without Gln supplementation during another 5 d chemotherapy course (*n* = 21)	Immunologic, nutrition, and anthropometric parameters after 5 d course of chemotherapy, stomatitis, and antibiotic use	Decreased lymphocyte count, increased complements 3 and 4, increased prealbumin and transferrin, no differences in anthropometric parameters, reduced stomatitis, and antibiotic use
Kuskonmaz et al. 2008 [177]	41 pediatric patients [mean age (y) ± SEM: 8.3 ± 5.2 (Gln) and 6.9 ± 4.3 (control)] who underwent HSCT	Case control	Parenteral Gln; IV Gln (0.4 g/kg/d) for ~13 d with or without PN (*n* = 21)	Controls were elected from presupplemented period and matched to cases with respect to donor type, diagnosis, and age	Mucositis (grades 3 and 4), PN use, neutrophil engraftment, incidence and duration of fever, documented infections, acute GVHD, sinusoidal obstruction syndrome, drug-related toxicity, LOS in hospital, and mortality	Nonsignificant trend toward reduced mucositis, sinusoidal obstruction syndrome and drug-related toxicity, reduced duration of fever, no differences in PN use, neutrophil engraftment, infections, acute GVHD, LOS in hospital or mortality, safe

BMT: bone marrow transplant; AA: amino acid; QID: 4 times a day; PN: parenteral nutrition; GVHD: graft versus host disease; LOS: length of stay; BID: twice a day; EN: enteral nutrition; SEM: standard error of the mean; HSCT: hematopoietic stem cell transplant; IV: intravenous.

**Table 6:** Glutamine in pediatric patients with severe burns/trauma.

Reference	Subjects	Design	Gln	Control	Outcomes	Results
Sheridan et al. 2004 [190]	7 pediatric patients aged 1.3–15.8 y with serious burns (≥20% of BSA) tolerating enteral feedings	Randomized double-blind crossover	Enteral Gln; EN (Pediasure and/or Jevity) with Gln (0.6 g/kg/d) replacing 20% of essential and nonessential AA for 48–72 h (*n* = 7)	Isocaloric isonitrogenous standard EN (Pediasure and/or Jevity) supplemented with modular protein (Promod) for 48–72 h (*n* = 7)	Whole-body protein kinetics (IV infusion of L-[1–13C]Leu, NaH13CO3) after 48 h enteral feeding (fed state), nitrogen balance, plasma Gln concentrations	Decreased Leu flux and Leu oxidation rate, no differences in net balance of Leu accretion into proteins, nitrogen balance or plasma Gln concentrations, well tolerated
Chuntrasakul et al. 2003 [195]	36 trauma patients aged 15–60 y (*n* = 16 severe trauma; *n* = 20 burn BSA 30–60%)	Randomized controlled	Enteral Gln; exclusive EN enriched with Arg, Gln, omega-3 fatty acids (Neomune) started on postinjury d-2 until d-10 (≤0.15 g/kg/d Gln) (*n* = 18)	Standard exclusive EN for trauma patients (traumacal) started on postinjury d-2 until d-10 (≤0.07 g/kg/d Gln) (*n* = 18)	Biochemical and immune parameters after 10 d supplementation, morbidity, and mortality	No differences in immunologic or biochemical parameters (except increased serum total protein, decreased serum triglycerides), no significant differences in nitrogen balance, LOS in ICU/hospital, d to wean off ventilator, or mortality, well tolerated
Yang and Xu, 2007 [196]	46 patients aged 7–68 y with severe traumatic brain injury	Randomized controlled	Enteral or parenteral Gln; Gln-Ala dipeptide (*n* = 23)	Routine nutritional therapy (*n* = 23)	LOS in neurosurgical ICU, mortality, lymphocyte count, and related complications	No significant differences in mortality, LOS in neurosurgical ICU, lung infection or alimentary tract hemorrhage or lymphocyte count

BSA: body surface area; EN: enteral nutrition; AA: amino acid; IV: intravenous; LOS: length of stay; ICU: intensive care unit.

**Table 7:** Enteral glutamine supplementation in chronic diseases of childhood.

Reference	Subjects	Design	Gln	Control	Outcomes	Results
Hankard et al. 1998 [204]	6 DMD boys aged 8–13 y	Time series	Study d-2: oral Gln (0.6 g/kg) dissolved in flavoured water (Kool-Aid) given over 5 h (*n* = 6)	Study d-1: oral flavoured water (Kool-Aid) given over 5 h (*n* = 6)	Whole body protein and Gln metabolism (IV infusion of L-[1–13C]Leu and L-[2–15N]Gln) in postabsorptive state (14 h fast) while drinking placebo on d-1 and Gln supplement on d-2, plasma AA concentrations	Decreased Leu release from protein breakdown and Leu oxidation rate, no effect on nonoxidative Leu disposal (an index of protein synthesis), increased whole-body Gln exchange in plasma, decreased Gln from protein degradation and Gln de novo synthesis, increased plasma Gln concentration, decreased plasma concentrations of essential AA (Leu, Phe, Lys)
Escolar et al. 2005 [206]	35 ambulant steroid-naive DMD boys aged 4–10 y	Randomized double-blind multicentre	Oral Gln (0.3 g/kg/d BID) for 6 mo (*n* = 19)	Placebo for 6 mo (*n* = 16)	Efficacy as assessed by changes at 6 mo in: (1) average manual muscle testing score, quantitative muscle-testing, (2) timed functional tests, pulmonary function tests, and safety	(1) No effect on manual or quantitative measurements of muscle strength, (2) young age (<7 y) subgroup showed less deterioration in timed functional tests, but no differences for entire cohort or for old age (≥7 y) subgroup, safe, and well tolerated
Mok et al. 2006 [205]	26 DMD boys aged 7–15 y	Randomized double-blind	Oral Gln (0.5 g/kg/d) given as powder mixed with yogurt for 10 d (*n* = 13)	Oral isonitrogenous nonspecific AA mixture given as powder mixed with yogurt for 10 d (*n* = 13)	Whole-body protein and Gln metabolism (IV infusion of L-[1–13C]Leu and L-[2–15N]Gln) in postabsorptive state after 10 d supplementation, body composition (BIA and 3-d urinary creatinine excretion), plasma concentrations of AA	Decreased rate of Leu appearance (an index of whole-body protein degradation) and endogenous Gln from protein degradation (both groups), no effect on Leu oxidation rate, nonoxidative Leu disposal (an index of protein synthesis), whole-body Gln exchange in plasma, Gln de novo synthesis or plasma Gln concentrations, no effect on fat-free mass, % fat mass, muscle mass or wt, safe, and well tolerated
Mok et al. 2009 [209]	30 ambulant DMD boys aged 2–10 y	Randomized double-blind crossover multicentre	Oral Gln (0.5 g/kg/d) for 4 mo (*n* = 30)	Oral placebo (maltodextrin) for 4 mo (*n* = 30)	Efficacy as assessed by changes at 4 mo in: (1) walking speed, (2) 2-minute walk test, work, power, muscle mass (urinary creatinine), myofibrillar protein breakdown (urinary 3-methyl-histidine/creatinine), serum creatine phosphokinase, % fat mass, fat-free mass (BIA), safety	No differences in measures of function: (1) walking speed, (2) 2-minute walk test, work or power, but steroid-treated subgroup showed less deterioration in functional measures during Gln phase, no differences in muscle mass, myofibrillar protein breakdown, or serum creatine phosphokinase, increased % fat mass, blunted increase in fat-free-mass, safe, and well tolerated,
Williams et al. 2004 [222]	27 children aged 5.2–17.9 y with sickle cell anemia	Case series	Oral Gln supplement (0.3 g/kg/d BID) for 24 wk	None	Measures of REE (indirect calorimetry/Harris Benedict equation) and other nutritional parameters after 24 wk supplementation	Decreased median REE overall with greater decrease in underweight (<90% IBW) subgroup, increased BMI, %FM and handgrip strength, increased plasma concentrations of Gln and Trp
Darmaun et al. 2004 [225]	9 prepubertal children aged 7–13 y with cystic fibrosis; undernourished (wt/height <50% ile) or short (height <5% ile)	Time series (order of Gln and rhGH regimens randomized)	4-wk supplementation with: (1) oral Gln (0.7 g/kg/d), (2) SC rhGH and (3) Gln + rhGH combined	Baseline	Whole-body protein and Gln metabolism (IV infusions of H13CO3Na, L-[1–13C]Leu and L-[2–15N]Gln) in postabsorptive state (after 12 h fast) after 4-wk treatment, body composition (skinfold thickness, BIA, DXA), plasma concentrations of glucose, hormones, growth factors, and AA	No effect on Leu release from proteolysis, Leu oxidation, or nonoxidative Leu disposal (an index of protein synthesis), no effect on Gln appearance rate, Gln release from proteolysis, or Gln de novo synthesis, increased plasma Gln concentrations, increased lean body mass, no effect on glucose metabolism
Mauras et al. 2010 [229]	10 type 1 diabetic adolescents on insulin pumps; mean ± SD age: 15.2 ± 1.4 y	Randomized double-blind crossover	Oral Gln drink before exercise and at bedtime (0.25 g/kg/dose) (*n* = 10)	Oral placebo drink (calorie and nitrogen free) before exercise and at bedtime (*n* = 10)	Blood glucose % drop from baseline during exercise, hypoglycemia during exercise, postexercise overnight hypoglycemia, cummulative probability of postexercise overnight hypoglycemia	During exercise, no differences in blood glucose % drop from baseline or proportion that develop hypoglycemia, increased postexercise overnight frequency of hypoglycemic events, increased cummulative probability of postexercise overnight hypoglycemia

DMD: Duchenne muscular dystrophy; IV: intravenous; AA: amino acid; BID: twice a day; BIA: bioelectrical impedance analysis; wt: weight; REE: resting energy expenditure; IBW: ideal body weight; BMI: body mass index; FM: fat mass; rhGH: recombinant growth hormone; SC: subcutaneous; DXA: dual X-ray absorptiometry.
